# Prevention of tuberculosis in macaques after intravenous BCG immunization

**DOI:** 10.1038/s41586-019-1817-8

**Published:** 2020-01-01

**Authors:** Patricia A. Darrah, Joseph J. Zeppa, Pauline Maiello, Joshua A. Hackney, Marc H. Wadsworth, Travis K. Hughes, Supriya Pokkali, Phillip A. Swanson, Nicole L. Grant, Mark A. Rodgers, Megha Kamath, Chelsea M. Causgrove, Dominick J. Laddy, Aurelio Bonavia, Danilo Casimiro, Philana Ling Lin, Edwin Klein, Alexander G. White, Charles A. Scanga, Alex K. Shalek, Mario Roederer, JoAnne L. Flynn, Robert A. Seder

**Affiliations:** 1 Vaccine Research Center, National Institute of Allergy and Infectious Diseases (NIAID), National Institutes of Health (NIH), Bethesda, MD USA; 2 Department of Microbiology and Molecular Genetics and Center for Vaccine Research, University of Pittsburgh School of Medicine, Pittsburgh, PA USA; 3 Ragon Institute of MGH, Harvard, and MIT, Cambridge, MA USA; 4 Department of Chemistry, Institute for Medical Engineering and Sciences (IMES), MIT, Cambridge, MA USA; 5 Broad Institute of MIT and Harvard, Cambridge, MA USA; 6 Department of Infectious Diseases and Microbiology, University of Pittsburgh School of Public Health, Pittsburgh, PA USA; 7 Aeras, Rockville, MD USA; 8 Department of Pediatrics, Children’s Hospital of the University of Pittsburgh of UPMC, Pittsburgh, PA USA; 9 Division of Animal Laboratory Resources, University of Pittsburgh School of Medicine, Pittsburgh, PA USA; 10 Koch Institute for Integrative Cancer Research, MIT, Cambridge, MA USA

**Keywords:** Immunological memory, Infection, Live attenuated vaccines

## Abstract

*Mycobacterium tuberculosis* (Mtb) is the leading cause of death from infection worldwide^1^. The only available vaccine, BCG (Bacillus Calmette–Guérin), is given intradermally and has variable efficacy against pulmonary tuberculosis, the major cause of mortality and disease transmission^1,2^. Here we show that intravenous administration of BCG profoundly alters the protective outcome of Mtb challenge in non-human primates (*Macaca mulatta*). Compared with intradermal or aerosol delivery, intravenous immunization induced substantially more antigen-responsive CD4 and CD8 T cell responses in blood, spleen, bronchoalveolar lavage and lung lymph nodes. Moreover, intravenous immunization induced a high frequency of antigen-responsive T cells across all lung parenchymal tissues. Six months after BCG vaccination, macaques were challenged with virulent Mtb. Notably, nine out of ten macaques that received intravenous BCG vaccination were highly protected, with six macaques showing no detectable levels of infection, as determined by positron emission tomography–computed tomography imaging, mycobacterial growth, pathology and granuloma formation. The finding that intravenous BCG prevents or substantially limits Mtb infection in highly susceptible rhesus macaques has important implications for vaccine delivery and clinical development, and provides a model for defining immune correlates and mechanisms of vaccine-elicited protection against tuberculosis.

## Abstract (web summary)

The delivery route and dose of the BCG vaccine profoundly alters the protective outcome after *Mycobacterium tuberculosis* challenge in non-human primates.

## Main

Two billion people worldwide are infected with Mtb, with 10 million new cases of active tuberculosis (TB) and 1.7 million deaths each year^1^. Prevention of pulmonary infection or disease in adolescents and adults would have the largest effect on the epidemic by controlling Mtb transmission^3^. The only licensed TB vaccine, BCG (live, attenuated *Mycobacterium bovis*), is administered intradermally at birth and provides protection against disseminated TB in infants but has variable efficacy against pulmonary disease in adolescents and adults^2^.

T cell immunity is required to control Mtb infection and prevent clinical disease^4^. A major hurdle to developing an effective and durable T-cell-based vaccine against pulmonary TB is to induce and sustain T cell responses in the lung to immediately control infection while also eliciting a reservoir of systemic memory cells to replenish the lung tissue. Intradermal and intramuscular administration—the most common routes of vaccine administration—do not induce high frequencies of resident memory T (T_RM_) cells in the lung. Studies performed 50 years ago suggested that administration of BCG by aerosol (AE) or intravenous (IV) routes enhanced protection in non-human primates (NHPs) challenged shortly after immunization^5–8^. However, there remains a limited understanding for mechanisms by which dose and route of BCG influence systemic and tissue-specific T cell immunity, and whether optimizing these variables would lead to high-level prevention of Mtb infection and disease. We hypothesized that a sufficiently high dose of IV BCG would elicit a high frequency of systemic and tissue resident T cells mediating durable protection against Mtb infection and disease in highly susceptible rhesus macaques.

## Experimental design and safety

**Extended Data Fig. 1:**
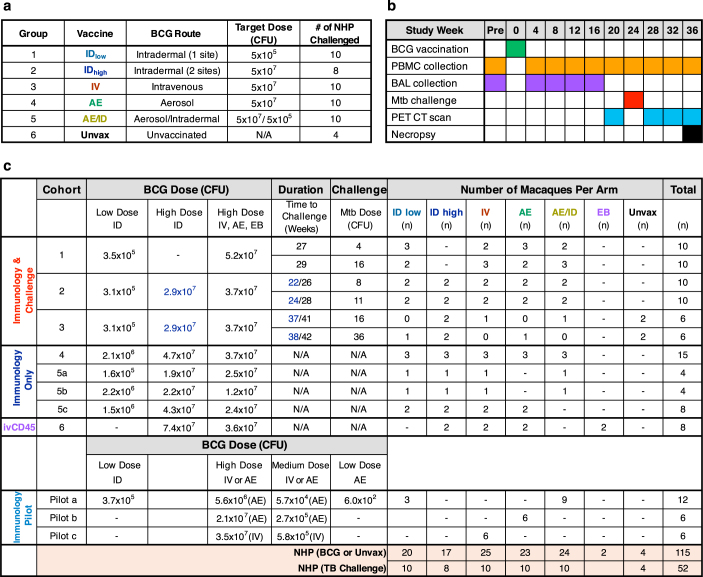
Study design, vaccine regimens, macaques and cohorts. **a**, Vaccine groups including route of BCG administration, target dose of BCG to be delivered (CFUs), and number of NHPs per BCG regimen (*n* = 10 macaques except ID_high_, *n* = 8 macaques). Note that ID_low_ and ID_high_ groups received BCG in one or two sites, respectively, and AE/ID group received AE (high-dose) and ID (low-dose) BCG simultaneously. Unvaccinated macaques (*n* = 4) were used as Mtb challenge controls. **b**, Timeline for Mtb challenge cohorts including weeks relative to BCG vaccination for PBMC and BAL sample collection, Mtb challenge, PET–CT scanning, and scheduled necropsy after challenge. Macaques that met humane end-point criteria were euthanized earlier than 12 weeks post-challenge (Supplementary Table 1). **c**, Data are from a total of 115 rhesus macaques, 52 of which were challenged with Mtb. Owing to the ABSL-3 capacity constraints and logistical limits in the number of macaques that can be sampled, scanned by PET–CT, or necropsied at any given time point, studies were broken into sequentially immunized and/or challenged cohorts. A maximum of 20 NHPs were infected with Mtb in any challenge cohort with infections split over 2 days, staggered by 2 weeks. The actual doses of BCG administered, determined by subsequent culture, is noted for each vaccine group. The time interval between vaccination and challenge is noted in weeks and the challenge dose of Mtb (CFUs) is listed for each challenge cohort (BCG vaccine dose and Mtb challenge dose for individual NHPs, along with peak immune responses and detailed outcome data, is provided in Supplementary Table 1). Protection data are from 8–10 BCG-immunized NHPs per group and 4 unvaccinated controls in cohorts 1–3 (‘Immunology & challenge’). Per protocol, BAL samples were not collected from animals 8 weeks before, or after, Mtb challenge. Three NHPs per vaccine group were immunized just as in cohorts 1–3 but were not challenged. Instead, these macaques (cohort 4; ‘Immunology only’) were sampled (BAL, PBMC) for 6 months after BCG immunization and then euthanized to perform extensive immune analysis in various tissues at what would have been the time of challenge. BAL samples from cohort 4 were transcriptionally profiled at weeks 13 and 25. Cohort 5 (a–c) includes 4 macaques per group (except AE and AE/ID groups, *n* = 2 NHPs each) that were immunized with BCG and were euthanized 1–3 months later to assess BCG CFUs and T cell responses in various tissues. NHPs in cohort 6 (‘ivCD45’, *n* = 2 macaques per group) received anti-CD45 injection before necropsy to distinguish blood- and tissue-derived cells. Pilot cohorts (a–c) include NHPs enrolled in the dose-finding pilot study (*n* = 3 macaques per dose and route; ‘Immunology pilot’).

**Extended Data Fig. 2:**
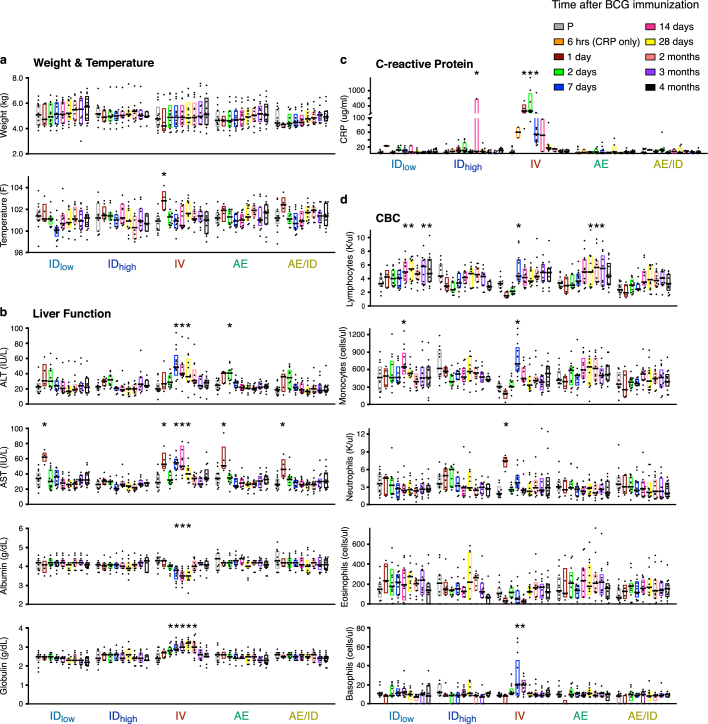
Clinical parameters after BCG vaccination in NHPs. To assess safety of BCG vaccinations, all macaques (cohorts 1–4 excluding unvaccinated) were monitored for changes in several clinical parameters at various time points after BCG. After vaccination, changes were observed predominantly in IV BCG macaques; however, all were transient. **a**, Weight and temperature: there was a 0.9 °C increase in body temperature in the IV BCG group at day 1, which resolved by day 2; the average pre-vaccination temperature across all NHPs was 38.4 °C. **b**, Liver function tests (alanine aminotransferase (ALT), aspartate aminotransferase (AST), albumin and globulin): there was a twofold increase in ALT and AST above pre-vaccination levels (20–30 IU l^−1^) in the IV BCG group, which resolved by day 28. **c**, C-reactive protein (CRP) in the IV BCG group increased up to a median of 400 μg ml^−1^ at day 2, which resolved by day 14; the average pre-vaccination CRP level in plasma across all NHPs ranged from 0 to 28 μg ml^−1^. **d**, Complete blood counts (CBC). Transient increases in numbers of circulating neutrophils (day 1) and lymphocytes, monocytes and basophils (day 7) were observed in the IV BCG group. All tests were performed longitudinally on whole blood at time of collection except CRP, which was batch-analysed from frozen plasma samples; the 6-h time point was measured for CRP only. Data points shown are individual NHPs (*n* = 11–13 per group, *n* = 63 total) with interquartile range (box) and median (line). For each parameter, pre-vaccination (P) measurements for all NHPs were combined and compared against distributions from every vaccine group at every time point using Dunnett’s test for multiple comparisons; *P < 0.05. No clinical signals, such as lethargy, appetite suppression or weight loss, were observed up to time of Mtb challenge, 24 weeks later. Source Data

The central aim of this study was to assess how the route and dose of BCG vaccination influence systemic and tissue-resident T cell immunity, and protection after Mtb challenge. Rhesus macaques were vaccinated with 5 × 10^7^ colony-forming units (CFUs) of BCG by intradermal (ID_high_), AE or IV routes, or with a combination of both AE (5 × 10^7^ CFUs) and ID (5 × 10^5^ CFUs; AE/ID) (Extended Data Fig. 1a). Immune responses and protective efficacy of these regimens were compared to the standard human dose given ID (5 × 10^5^ CFUs; ID_low_). The dose of BCG selected for AE and IV vaccine groups was based on pilot dose-ranging studies (Supplementary Data 1). After BCG vaccination, immune responses in blood and bronchoalveolar lavage (BAL) were assessed over 24 weeks, after which NHPs were challenged with a low dose of Mtb (Extended Data Fig. 1b). Other macaques in each group were euthanized 1 or 6 months after vaccination for immune analysis of tissue responses (Extended Data Fig. 1c). To assess safety of BCG vaccinations, several clinical parameters were measured and found to be transiently affected by only IV BCG (Extended Data Fig. 2). A summary of all NHPs in this study and doses of BCG and Mtb administered are provided in Extended Data Fig. 1c and Supplementary Table 1.

## Cellular composition of BAL and blood

**Fig. 1:**
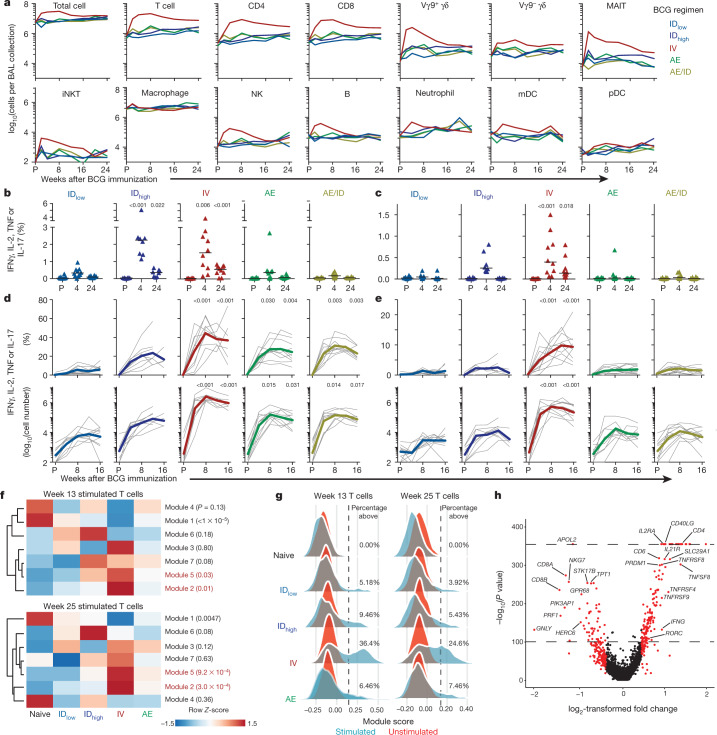
Cellular composition and immune analysis in blood and BAL after BCG vaccination. **a**, Number of cells (geometric mean) per BAL collection for leukocyte populations in each vaccine group before (pre, P) and up to 24 weeks after BCG; Supplementary Data 2 shows individual NHPs and statistical comparisons. Data are from cohorts 1–4 (*n* = 11–13 macaques per group as outlined in Extended Data Fig. 1) except at weeks 2, 20 and 24 (cohort 4 only, *n* = 3). Vγ9^+/−^, Vγ9^+/−^ γδ T cells; MAIT, mucosal-associated invariant T cells; mDC, myeloid dendritic cells; NK, natural killer cells; iNKT, invariant natural killer cells; pDC, plasmacytoid dendritic cells. **b**, **c**, Percentage of memory CD4 (**b**) or CD8 (**c**) T cells in PBMCs producing IFNγ, IL-2, TNF or IL-17 after PPD stimulation in vitro. Shown are individual and median (horizontal bar) responses for NHPs in challenge study (cohorts 1–3, *n* = 8–10 macaques) at weeks 4 (peak) and 24 (time of challenge) after BCG vaccination. **d**, **e**, Percentage (top) and number (bottom) of cytokine^+^ memory CD4 (**d**) and CD8 (**e**) T cells in the BAL before and up to 16 weeks after BCG vaccination. Shown are individual (grey lines) and mean (coloured lines) responses for challenge cohorts (*n* = 8–10 macaques). Each group was compared to ID_low_ at weeks 4 and 24 for PBMCs (one-way ANOVA; *P* values are Dunnett’s multiple comparison test) or weeks 8 and 16 for BAL (Kruskal–Wallis test; *P* values are Dunn’s multiple comparison test). **f**–**h**, Single-cell transcriptional analysis of BAL cells at weeks 13 and 25 after BCG vaccination (cohort 4; *n* = 3 per group). **f**, *Z*-scored heat maps of the average cellular score for modules identified in week 13 PPD-stimulated T cells at weeks 13 and 25 after BCG vaccination. Red *P* values indicate modules uniquely elevated in the IV BCG group (one-way ANOVA). **g**, Distributions of module 2 expression in unstimulated and stimulated T cells at weeks 13 and 25 for each group. Percentage module 2-positive is shown; positivity (dashed line) defined as 2 s.d. above the mean score of the unvaccinated (Naive) NHPs. **h**, Volcano plot showing differentially expressed genes between T cells positive and negative for module 2 at week 13 (*P* values calculated using the likelihood ratio test with Bonferroni correction). Source Data

**Extended Data Fig. 3:**
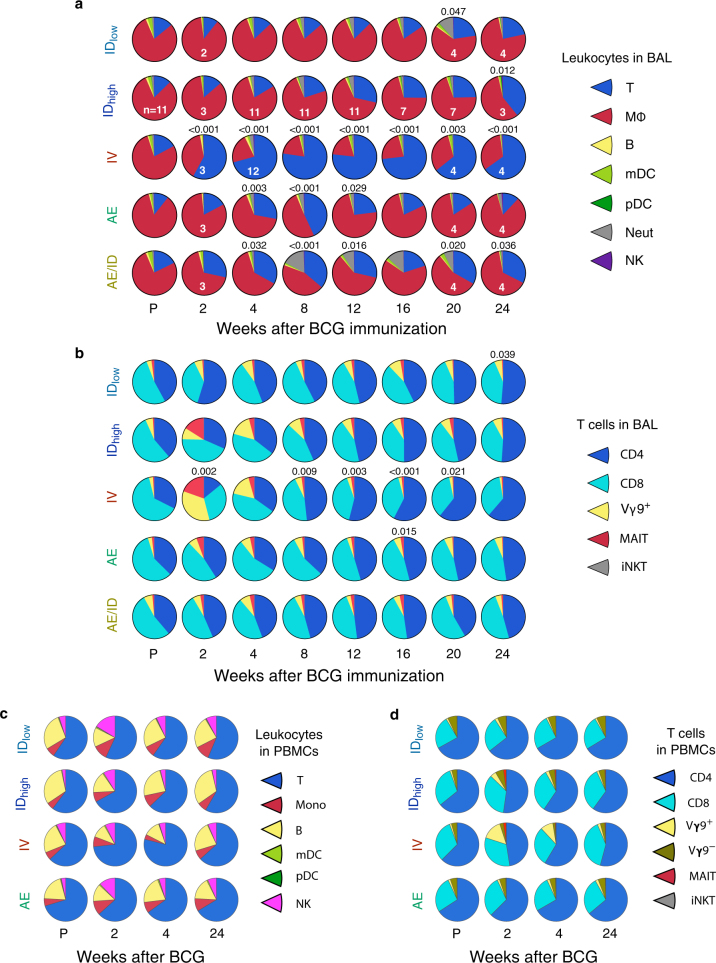
Proportions of leukocyte and T cell subsets in the BAL and PBMCs after BCG immunization. **a**–**d**, We assessed whether the composition of leukocytes in the BAL or PBMCs was altered after BCG vaccination. Shown are pie graphs comprising proportions of indicated leukocytes (**a**, **c**) or CD3^+^ T cell subsets (**b**, **d**) in BAL (**a**, **b**) and PBMCs (**c**, **d**) for each BCG regimen from pre-vaccination up to 24 weeks post-BCG, identified using multi-parameter flow cytometry as in Supplementary Data 8. **a**, In the BAL, the rapid and sustained increase in T cell (but not macrophage) number (Fig. 1a and Supplementary Data 2b) altered the overall cellular composition of BAL from approximately 75% alveolar macrophages (red) and 15% T cells (blue) before vaccination to approximately 65% T cells and 30% macrophages, even 6 months after IV BCG. **b**, To delineate the composition of BAL T cells further, the proportions of CD4 and CD8 T cells, as well as non-classical T cells (γδ, MAIT and iNKT) that may also have a role in protection against TB^9–11^ were assessed. Two weeks after vaccination, there was a substantial but transient increase in the proportion of Vγ9^+^ γδ T cells and MAIT cells after IV BCG, and a trend towards increased Vγ9^+^ γδ T cells and MAIT cells after BCG ID_high_. However, by 8 weeks, the proportions of these non-classical T cells contracted to pre-vaccination levels. **c**, **d**, A similar analysis was performed to determine how the route of BCG immunization influenced the composition of leukocytes in PBMCs. Here, IV BCG induced a transient increase in Vγ9^+^ γδ T cells but not MAIT cells. BAL pie graphs represent the average proportions from 13 NHPs per BCG regimen (cohorts 1–4; Extended Data Fig. 1c) except where indicated (white numbers in **a** also apply to **b**). PBMC pie graphs represent the average proportions from three NHPs per BCG regimen (cohort 4). B, B cells; Mφ, macrophages; Mono, monocytes; T, T cells; Neut, neutrophils. *P* values indicate differences compared to pre-vaccination within the same vaccine group using a Permutation test. Source Data

Because generating immune responses in the lung was a major focus of the study, we first assessed whether the BCG vaccination regimen altered the number or composition of leukocytes in the BAL. Only IV BCG vaccination elicited significant changes in BAL cell numbers: a 5–10-fold increase in total cells, accounted for largely by conventional T cells (Fig. 1a and Supplementary Data 2a, b). This resulted in a sustained inversion of the alveolar macrophage:T-cell ratio up to 6 months after IV BCG vaccination (Extended Data Fig. 3a). Non-classical T cells (MAIT and Vγ9^+^ γδ) that can contribute to protection against TB^9–11^ were transiently increased 2–4 weeks after IV BCG (Fig. 1a, Extended Data Fig. 3b and Supplementary Data 2b). A similar analysis performed on peripheral blood mononuclear cells (PBMCs) showed no significant changes in leukocyte composition (Extended Data Fig. 3c, d). Neither BAL nor PBMCs exhibited changes in the proportion of natural killer cells, which were recently suggested to correlate with protection^12,13^ (Extended Data Fig. 3a, c). Finally, there were no increases in cytokines associated with trained innate immunity^14,15^ in stimulated PBMCs after ID or IV BCG immunization (Supplementary Data 3). Overall, these data show that IV BCG immunization, in contrast to AE or ID, results in significant and sustained recruitment of T cells to the airways and substantially alters the ratio of T cells to macrophages.

## Antigen-responsive adaptive immunity

**Extended Data Fig. 4:**
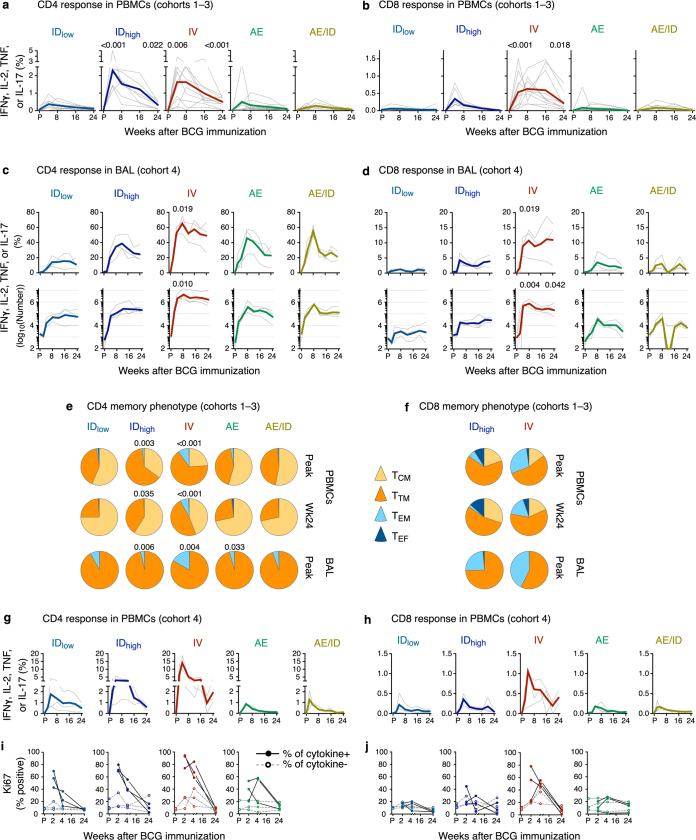
Extended immune data from challenge and immunology cohorts. **a**, **b**, Full kinetics of PBMC responses from NHPs in challenge cohorts (cohorts 1–3, *n* = 8–11 macaques) as in Fig. 1b, c. Shown is the frequency of memory CD4 (**a**) or CD8 (**b**) T cells producing any combination of IFNγ, IL-2, TNF or IL-17 in response to PPD stimulation at various time points before and up to 24 weeks after BCG. Grey lines are individual NHP responses; bold, coloured lines represent the median response. Each group was compared to ID_low_ at weeks 4 and 24 (one-way ANOVA; *P* values are Dunnett’s multiple comparison test). **c**, **d**, T cell responses from a replicate cohort of similarly BCG-immunized rhesus macaques (cohort 4, *n* = 3 NHPs) from which BAL was collected for 24 weeks after BCG vaccination. Shown is the frequency (top) or absolute number (log_10_-transformed; bottom) of CD4 (**c**) or CD8 (**d**) memory T cells expressing any combination of IFNγ, IL-2, TNF or IL-17 in response to PPD stimulation, before and up to 24 weeks after BCG vaccination. Kruskal–Wallis test was used to compare each group to ID_low_ at weeks 8 (peak) and 24 (time of challenge); *P* values are Dunn’s multiple comparison test. **e**, **f**, The memory phenotype of antigen-responsive CD4 (**e**) and CD8 (**f**) T cells in PBMCs and BAL at the peak of the response (week 4 for PBMC, week 8–12 for BAL; cohorts 1–3, *n* = 8–10 macaques) and time of challenge (week 24 collected for PBMC only) was assessed. Cytokine-positive T cells from PBMCs were categorized as central memory (T_CM_), T_TM_, effector memory (T_EM_), or terminal effectors (T_EF_) based on expression of CD45RA, CD28 and CCR7 as shown in Supplementary Data 10. Most responding cells in PBMCs were central memory and transitional memory T cells, with the proportion of transitional memory cells greater in ID_high_- and IV-BCG-immunized NHPs compared with the ID_low_ group. In BAL, where T cells are CCR7-negative, most responding CD4 T cells were CD45RA^−^CD28^+^ T_TM_ cells (Supplementary Data 9). For CD8 memory phenotypes, pie graphs are shown only for groups that displayed measurable frequencies of cytokine^+^ CD8 T cells. IV-BCG-immunized NHPs had larger proportions of T_EM_ cells in PBMCs and BAL, which suggests a more diverse composition of memory and effector cells than other routes. *P* values indicate differences compared to ID_low_ using a permutation test (CD4 pie graphs only). **g**–**j**, PBMC T cell responses from a replicate cohort of similarly BCG-immunized rhesus macaques (cohort 4, *n* = 3 macaques). Shown is the frequency of CD4 (**g**) and CD8 (**h**) memory T cells producing any combination of IFNγ, IL-2, TNF or IL-17 in response to PPD stimulation before and up to 24 weeks after BCG. **i**, **j**, As an immunological indicator of recent antigen exposure and proliferation due to BCG persistence in vivo, Ki-67 expression in PBMCs over the course of immunization was assessed. Shown is the percentage of cytokine-positive (closed symbols, solid lines) or cytokine-negative (open symbols, dashed lines) memory CD4 or CD8 T cells expressing Ki-67 as identified in Supplementary Data 11. In IV-BCG-immunized NHPs, at least 60% of antigen-responsive CD4 T cells in blood were Ki-67^+^ at 2 and 4 weeks after BCG but were at baseline 6 months later. Source Data

We next evaluated how these regimens influenced the ability of T cells responsive to mycobacterial antigen (such as purified protein derivative (PPD)) to produce the canonical cytokines (IFNγ, IL-2, TNF or IL-17) that are important for protection against TB^4,16,17^. At the peak of the PBMC response (week 4), cytokine-producing CD4 T cells were higher in NHPs immunized with ID_high_ or IV BCG compared with those immunized with ID_low_ BCG; these responses declined over time but remained increased at week 24 (time of challenge; Fig. 1b and Extended Data Fig. 4a, g). PBMC CD8 responses in IV-immunized NHPs were greater than ID_low_ NHPs at both time points (Fig. 1c and Extended Data Fig. 4b, h). In BAL, antigen-responsive T cells peaked at 8 weeks and were largely maintained until time of challenge (Fig. 1d, e and Extended Data Fig. 4c, d). Compared with ID_low_ BCG, ID_high_ or AE BCG immunization elicited tenfold more PPD-responding CD4 T cells in BAL; IV BCG elicited 100-fold more PPD-responsive CD4 T cells, with approximately 40% of cells responding (Fig. 1d). Furthermore, only IV BCG induced an increase in antigen-responsive CD8 T cells (Fig. 1e). Central memory and transitional memory (T_TM_) T cells^18^ comprised the majority of CD4 T cell responses in PBMCs across all vaccine groups at the peak of the response, whereas T_TM_ cells predominated in the BAL (Extended Data Fig. 4e, f). IV-BCG-vaccinated NHPs had the largest proportion of T_TM_ cells in PBMCs and effector memory (T_EM_) cells in BAL.

**Extended Data Fig. 5:**
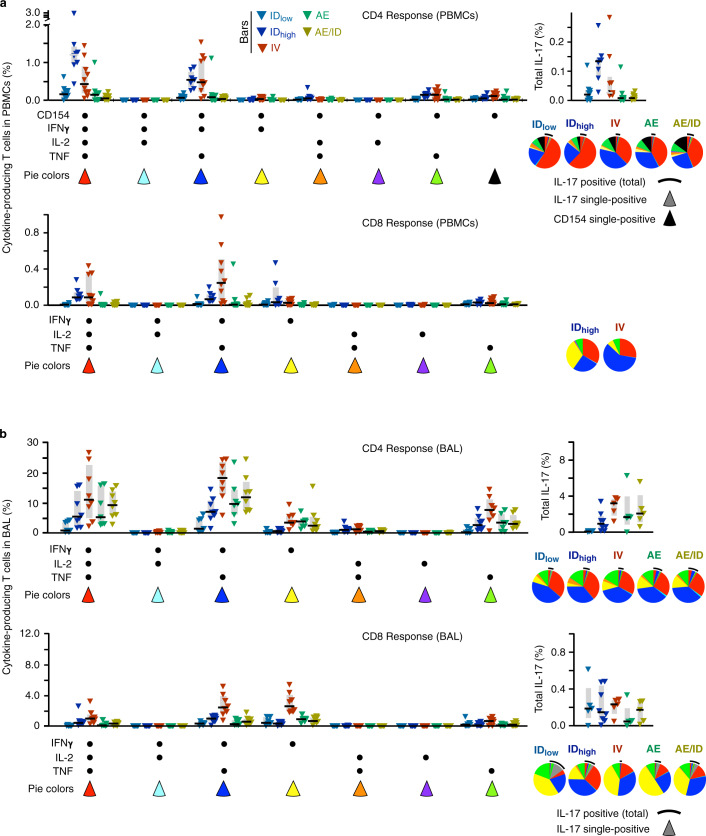
Quality of T cell responses in PBMCs and BAL after BCG immunization. The composition of the cytokine responses at the single-cell level, or ‘quality’ of the response, can reveal distinct functional differences that associate with protection against Mtb and other pathogens^19,20^. Here, the quality was defined by the relative proportion of antigen-stimulated cells producing every combination of IFNγ, IL-2 and TNF, with or without CD154 or IL-17. CD154 (also known as CD40L) expression in PBMCs was measured as a sensitive marker for detection of all antigen-stimulated CD4 T cells^21^ based on evidence for CD4-dependent, IFNγ-independent mechanisms of protection against TB^22,23^. Shown are peak PPD-responsive memory CD4 and CD8 T cell responses in PBMCs (**a**, week 4) or BAL (**b**, week 12) after BCG vaccination for challenge cohorts 1–3 (*n* = 8–11 NHPs); analysis of all time points is shown in Supplementary Data 4 and 5. **a**, Bar graphs show the frequency of T cells in PBMCs expressing CD154 with IFNγ, IL-2, or TNF production, and total IL-17 production (CD4 response, top) or IFNγ, IL-2, or TNF for the CD8 response (bottom). Individual NHP responses are shown with interquartile range (bar) and median (horizontal line). Pie graphs represent the proportion of the total response comprising each cytokine combination, averaged for all NHPs, and are not shown for groups with low to undetectable responses. The proportion of the response producing IL-17 (with or without other cytokines) is indicated with a black arc and the proportion expressing CD154 alone is the black pie section. **b**, Bar graphs show the frequency of CD4 or CD8 T cells in BAL producing IFNγ, IL-2 or TNF, and total IL-17 production. Pie graphs represent the average proportion of total cytokine production comprising each cytokine combination; the proportion of the total response producing IL-17 (with or without other cytokines) is indicated with a black arc. Despite the notable differences in the magnitude of responses amongst BCG regimens, there were no differences in the quality of CD4 T cell responses nor CD8 T cell responses in PBMC or BAL. Of note, approximately 90% of the CD4 T cell responses were composed of T_H_1 cytokines with fewer than 10% also producing IL-17; most IL-17 producing CD4 T cells co-expressed T_H_1 cytokines. Source Data

Despite differences in the magnitude of T cell responses among vaccine regimens, there were no differences in the quality of T cell responses (that is, the proportion of cells producing each combination of IFNγ, IL-2, TNF and IL-17)^19,20^ in PBMCs (Extended Data Fig. 5a and Supplementary Data 4) or the BAL (Extended Data Fig. 5b and Supplementary Data 5). Of the CD4 T cell responses, 90% consisted of T helper 1 (T_H_1) cytokines, with fewer than 10% also producing IL-17; most IL-17-producing CD4 T cells co-expressed T_H_1 cytokines (Extended Data Fig. 5). Notably, approximately 10% of antigen-responsive CD4 T cells in PBMCs expressed CD154^21^ but no T_H_1 or T_H_17 cytokines (Extended Data Fig. 5a and Supplementary Data 4), which suggests that there may be underlying qualitative differences among vaccine group responses that are not measured by the canonical T cell cytokines commonly used to assess BCG-elicited immunity^22,23^.

**Extended Data Fig. 6:**
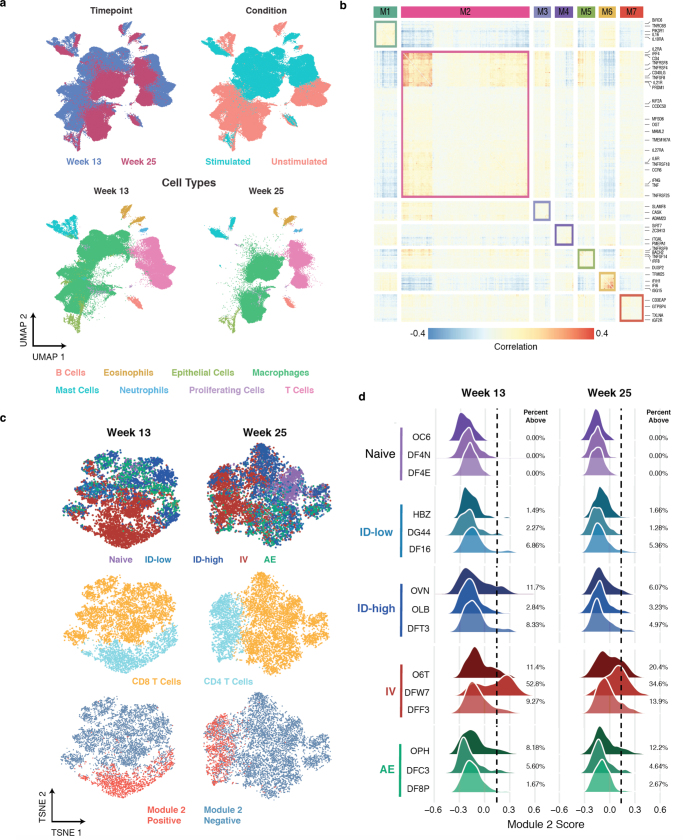
Identification of gene modules and distribution of module scores. A total of 162,490 single-cell transcriptomes derived from unstimulated and PPD-stimulated BAL cells from 15 NHPs (cohort 4, *n* = 3 per group) at weeks 13 (peak of BAL response) and 25 (time of challenge) were profiled. **a**, Uniform manifold approximation and projection (UMAP) plots of BAL cells at weeks 13 and 25 after BCG immunization, coloured by time point (top left), PPD stimulation condition (top right), and cell type (week 13, bottom left; week 25, bottom right). **b**, Gene–gene correlation heat map showing significant gene modules (M1–M7; top) identified among week 13 stimulated BAL T cells with select genes (right) highlighted. **c**, *t*-Distributed stochastic neighbour embedding (*t*-SNE) plots of stimulated BAL T cells from weeks 13 (left) and 25 (right), coloured by vaccine group (top), T cell subtypes (middle), and module 2-positivity (bottom). **d**, Histograms of the distribution of module 2 scores by vaccine group (colour) and macaque. Dashed line (placed at two s.d. above the mean score in the naive controls) indicates the threshold used to call cells as positive for the module. The percentage module 2-positive is shown for each NHP. Source Data

To expand the qualitative analysis of BAL T cell responses using an orthogonal approach, we performed single-cell mRNA sequencing (scRNA-seq) with Seq-Well^24^ to comprehensively assess phenotypic and transcriptional states among T cells that might underlie protective vaccine responses (Fig. 1f–h, Extended Data Fig. 6 and Supplementary Data 6). We examined correlated patterns of gene expression within unstimulated and PPD-stimulated T cells from BAL to identify groups of genes for which the coordinated activity differed by regimen (Extended Data Fig. 6b). A total of seven significant T cell modules were identified among in vitro-stimulated T cells 13 weeks after immunization (Supplementary Table 2) and used to generate expression scores across all T cells at weeks 13 and 25. Among these, we identified a stimulation-inducible module of gene expression, module 2, enriched for memory T cell functionality (Supplementary Table 3 and Methods), primarily expressed in a population of BAL CD4 T cells from IV-BCG-immunized NHPs at week 13, and maintained until week 25 (Fig. 1f, g, Extended Data Fig. 6c, d and Supplementary Table 2). Differential gene expression analysis, comparing T cells positive and negative for module 2 (Fig. 1h and Supplementary Table 4), showed enrichment of genes previously associated with protection against TB including *IFNG*, *TBX21*, *RORC*, *TNFSF8*^25^ and *IL21R*^26^.

**Extended Data Fig. 7:**
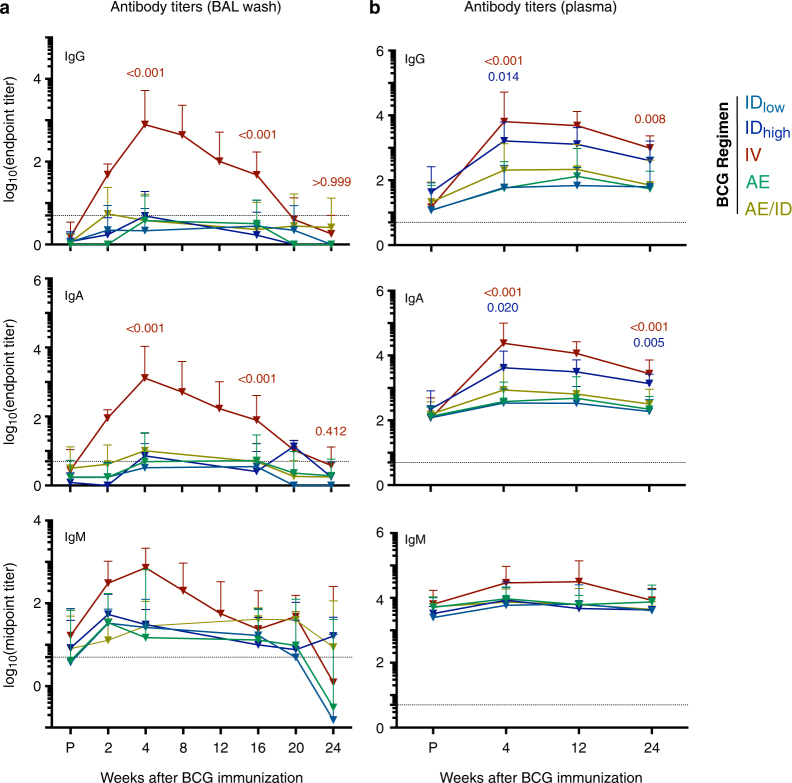
Humoral immune response in BAL and plasma after BCG immunization. Mtb-responsive antibody responses were assessed in BAL and plasma after BCG immunization. Mtb WCL-specific IgG, IgA and IgM antibody titres were measured from individual NHPs at various time points before and after BCG immunization. Shown are end-point titres for IgG and IgA and mid-point titres for IgM (in which the end point was not reached) **a**, Antibody titres in tenfold-concentrated BAL fluid (cohorts 1–4, *n* = 11–13 macaques except at weeks 2, 20 and 24, cohort 4 only, *n* = 3 macaques). In concentrated BAL fluid, antigen-responsive IgG, IgA and IgM were detected only in IV-BCG-immunized NHPs and returned to pre-vaccination levels by the time of challenge. **b**, Antibody titres in plasma (*n* = 11–13 macaques). In plasma, both ID_high_ and IV BCG elicited increased IgG and IgA antibody responses compared to ID_low_ BCG. Data are geometric mean and s.d.; dashed line indicates assay limit of detection. A Kruskal–Wallis test was used to compare all vaccine groups to ID_low_ at weeks 4, 16 and 24 (BAL) or weeks 4 and 24 (plasma); *P* values are from Dunn’s multiple comparison test (colour-coded to vaccine). Source Data

To further analyse adaptive immunity, we found that IV BCG elicited higher antibody responses in the BAL and plasma than the other routes. Mtb-specific IgG, IgA and IgM peaked 4 weeks after IV BCG vaccination and returned to baseline by 24 weeks in the BAL (Extended Data Fig. 7).

## *M. tuberculosis* challenge outcome

**Fig. 2:**
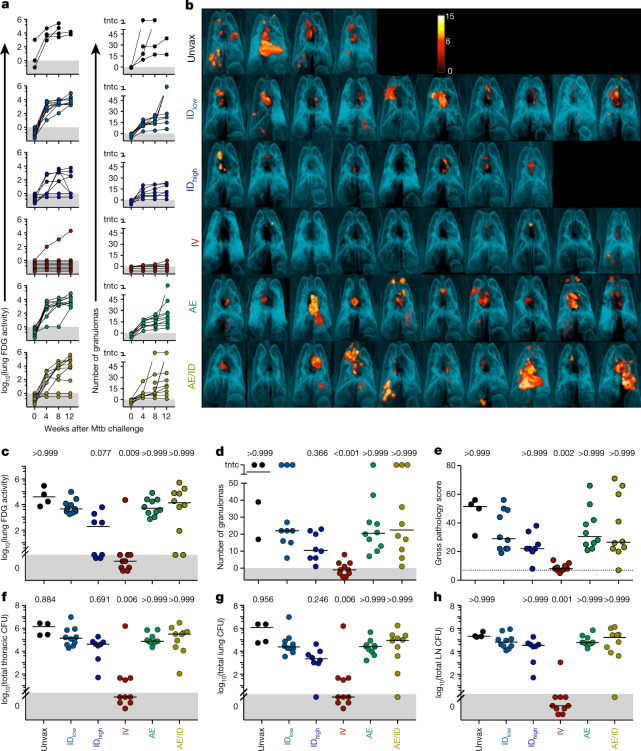
Protection against Mtb infection after IV BCG immunization. **a**, Lung inflammation (total FDG activity) and number of lung granulomas over the course of infection as measured by serial PET–CT scans. Each line shows one NHP over time; 3 NHPs (2 unvaccinated (unvax) and 1 ID_low_) reached a humane end point before 12 weeks. tntc, too numerous to count. **b**, Three-dimensional volume renderings of PET–CT scans of each NHP at the time of necropsy. PET was limited to the thoracic cavity; the standardized uptake value colour bar is shown in the top right and indicates FDG retention, a surrogate for inflammation. **c**–**h**, Total lung FDG activity (**c**), number of lung granulomas (**d**), gross pathology score (**e**), total thoracic CFUs (mycobacterial burden) (**f**), total lung CFUs (**g**) and total thoracic LN CFUs (**h**) at time of necropsy. Dashed line in **e** is assumed normal pathology score accounting for variability in LN size in healthy rhesus macaques. **c**–**h**, Symbols represent individual challenged macaques (cohorts 1–3, *n* = 8–10 vaccinated NHPs; *n* = 4 unvaccinated NHPs) and horizontal bars represent the median; all data points within the grey areas are zero. Kruskal–Wallis tests were used and reported *P* values represent Dunn’s multiple comparison test comparing each group to the ID_low_ group. Source Data

Six months after BCG immunization, NHPs were challenged in three separate cohorts with a nominal dose of 10 CFUs of the highly pathogenic Mtb Erdman strain, with a pre-defined study end point of 12 weeks after challenge (Extended Data Fig. 1b, c and Supplementary Table 1). Infection and disease were tracked serially using ^18^F-fluorodeoxyglucose (FDG) positron emission tomography–computed tomography (PET–CT) imaging. Total FDG activity in lungs, a measure of cellular metabolism that correlates with total thoracic mycobacterial burden^27,28^, was negative in all immunized macaques before Mtb challenge, but was increased throughout infection in unvaccinated NHPs (Fig. 2a). Three-dimensional reconstructions of pre-necropsy PET–CT scans are shown in Fig. 2b. All ID_low_- and AE-BCG-immunized NHPs had increased FDG activity in lungs over 12 weeks. Two NHPs in the ID_high_ and AE/ID BCG groups had no lung FDG activity and two NHPs in the ID_high_ group had inflammation at 8 weeks that returned to baseline by 12 weeks, suggesting partial protection. By contrast, nine out of ten IV-BCG-immunized NHPs had no lung FDG activity throughout the challenge phase (Fisher’s exact test, *P* < 10^−4^ compared to ID_low_ BCG) (Fig. 2a–c).

**Extended Data Fig. 8:**
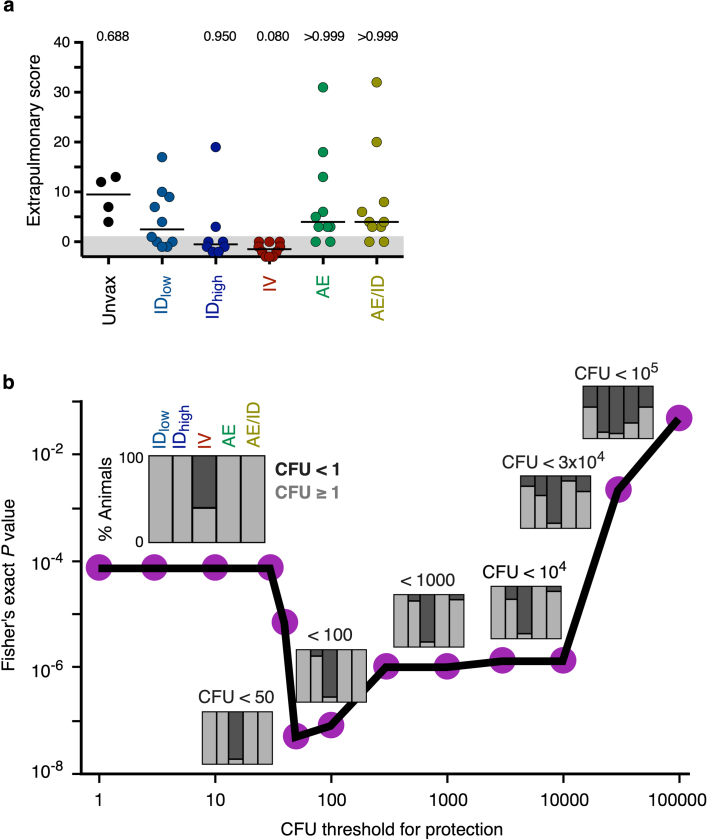
IV BCG protects against extrapulmonary disease and lung infection across a wide range of thresholds of protection. **a**, Extrapulmonary disease was scored at necropsy based on a published system^27^ taking into account the presence of Mtb-related pathology and Mtb growth from sites outside the thoracic cavity. Each symbol represents an animal and horizontal bars represent the median. Kruskal–Wallis tests were used and reported *P* values represent Dunn’s multiple comparison test comparing each group to ID_low._ All data points within the grey areas are zero. There was no extrapulmonary disease in any of the IV-BCG-vaccinated macaques, whereas the other groups had variable extrapulmonary involvement. **b**, Fisher’s exact test *P* values are plotted for a range of CFU thresholds evaluating protection. For each threshold, a stacked bar plot indicates the percentage of NHPs with fewer CFUs than the threshold (that is, protected), in each vaccine group. Immunization route significantly (*P* < 10^−4^) impacted protection at any given CFU threshold between <1 (sterile) and <10^4^. Source Data

PET–CT was used to track granuloma formation after Mtb infection as a correlate of active disease^27^. By 4 weeks and throughout infection, granulomas were detected in all unvaccinated as well as ID_low_-, ID_high_-, AE- and AE/ID-BCG-immunized NHPs (Fig. 2a). By contrast, IV-BCG-immunized NHPs had fewer granulomas compared with the benchmark ID_low_ BCG regimen (*P* < 0.001), with six out of ten NHPs having no granulomas throughout infection (Fig. 2a, d). Detailed necropsies showed that the IV-BCG-immunized group had lower gross pathology scores^27^ (Fig. 2e) compared with the ID_low_ BCG group (*P* = 0.002) and was the only group without detectable extrapulmonary disease (Extended Data Fig. 8a).

The primary measure of protection was a comprehensive quantification of Mtb burden (CFUs) at necropsy. The median total thoracic CFUs for ID_low_ BCG (5.1 ± 1.3, median ± interquartile range of log_10_-transformed total CFUs) was slightly lower than that of unvaccinated NHPs (5.9 ± 1.0 log_10_-transformed CFUs), consistent with ID_low_ BCG having a minimal protective effect in rhesus macaques (Fig. 2f). By contrast, the median total thoracic CFUs in IV-BCG-immunized NHPs was 0 (± 16 CFUs)—a more than 100,000-fold reduction compared with ID_low_ BCG (*P* = 0.006). Six out of ten IV-BCG-immunized macaques had no detectable Mtb in any tissue measured, and another three macaques had ≤45 total CFUs, all contained within one granuloma. Only one of ten IV BCG NHPs was not protected, with CFU values similar to ID_low_ NHPs (Fig. 2f). The ID_high_, AE and AE/ID groups had bacterial burdens similar to ID_low_ BCG.

Total thoracic bacterial burden can be separated into lung (Fig. 2g) and thoracic lymph node (LN) (Fig. 2h) CFUs. Only the IV BCG group was lower than the ID_low_ BCG group (lung, *P* = 0.006; LNs, *P* = 0.001), with nine of ten NHPs having no Mtb-positive LNs (Fig. 2h).

Protection can be defined as having less than a given number of total thoracic Mtb CFUs. By this criterion, protection was highly significant (Fisher’s exact test, *P* < 10^−4^) at any given threshold less than 10,000 CFUs (Extended Data Fig. 8b), with the IV BCG group showing 90% protection (95% confidence interval: 60–98%) at a threshold as low as 50 CFUs. Thus, BCG IV confers an unprecedented degree of protection in a stringent NHP model of TB.

## Immune responses after Mtb challenge

**Extended Data Fig. 9:**
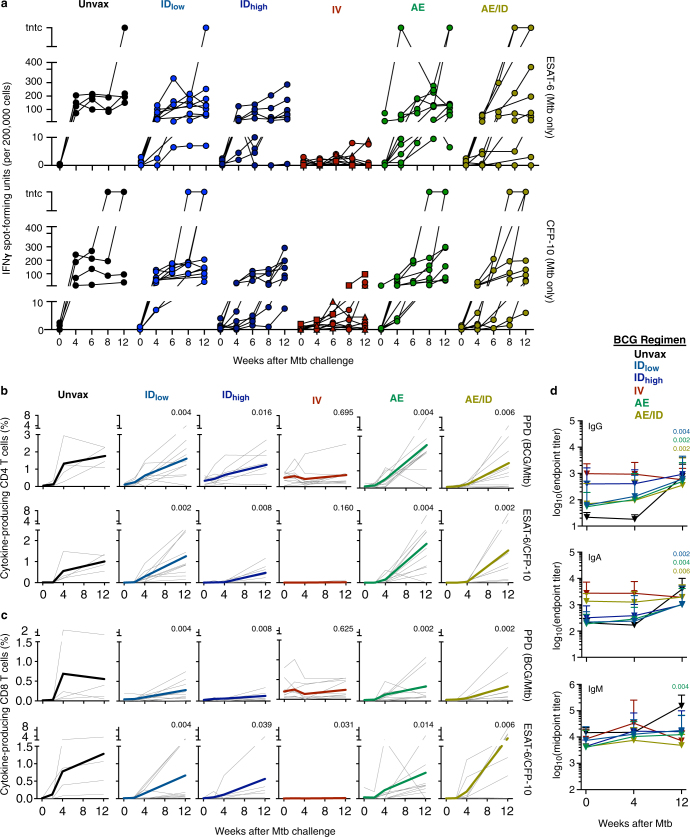
Post-challenge immune responses to mycobacterial antigens. **a**, PBMC response to ESAT-6 or CFP-10 peptides (antigens present in Mtb but not BCG) as determined by IFNγ ELISpot throughout Mtb infection. Each line is one NHP over time (*n* = 8–10 macaques; *n* = 4 unvaccinated); sterile animals are represented by a triangle, and non-sterile, protected animals (with 1 ≤ CFUs ≤ 50) denoted by squares. After infection, most animals in the AE or ID vaccine groups developed ESAT-6 or CFP10 ELISpot responses, which reflects a primary response to Mtb. By contrast, responses in the IV BCG group were lower than in the ID_low_ group at every time point after infection for ESAT-6 (4 weeks, *P* = 0.001; 6 weeks, *P* = 0.045; 8 weeks, *P* = 0.025; 12 weeks, *P* = 0.006) and CFP-10 (4 weeks, *P* < 0.0001; 6 weeks, *P* = 0.035; 8 weeks, *P* = 0.001; 12 weeks, *P* = 0.004). Kruskal–Wallis test was run at each time point with Dunn’s adjusted *P* values reported accounting for comparisons of all groups against ID_low_. **b**, **c**, The frequency of memory CD4 (**b**) and CD8 (**c**) T cells in PBMCs from BCG-immunized NHPs (*n* = 8–10) producing any combination of IFNγ, IL-2, TNF or IL-17 in response to stimulation with either PPD (antigen present in BCG and Mtb; top row) or pooled ESAT-6 and CFP-10 peptides (antigens present in Mtb only; bottom row) were measured at the time of challenge (0), and at 4, 8 and 12 weeks after Mtb challenge. Measurements from four unvaccinated, infected NHPs are included as controls (Unvax, black). Grey lines represent the responses of individual animals and bolded, coloured lines are the mean responses for each vaccine group. **d**, Antibody responses post-challenge. Mtb WCL-specific IgG, IgA and IgM antibody titres were measured in the plasma of unvaccinated (*n* = 4) and vaccinated (*n* = 8–10) NHPs at the time of challenge (0), and at 4 and 12 weeks after challenge. In **b**–**d**, Wilcoxon signed-rank unadjusted *P* values compare cytokine frequencies or antibody titres at week 12 after Mtb (or necropsy) to the time of challenge (week 0) within each vaccine group. Source Data

Measuring immune responses after challenge informs whether vaccine-elicited responses are boosted (anamnestic), and if de novo (primary) responses are generated to antigens expressed by the challenge microorganism (but not the vaccine). T cell responses to ESAT-6 and CFP-10—proteins expressed in Mtb but not BCG—are used to detect primary Mtb infection, even in BCG-immunized individuals. Peripheral T cell and antibody responses to these Mtb-specific antigens and those expressed by both BCG and Mtb (for example, PPD), were assessed after Mtb challenge (Extended Data Fig. 9). In contrast to all other groups, IV-BCG-immunized NHPs had low to undetectable primary or anamnestic T cell and antibody responses after TB infection, which suggests rapid elimination of Mtb after challenge.

## BCG and immune responses in tissues

**Fig. 3:**
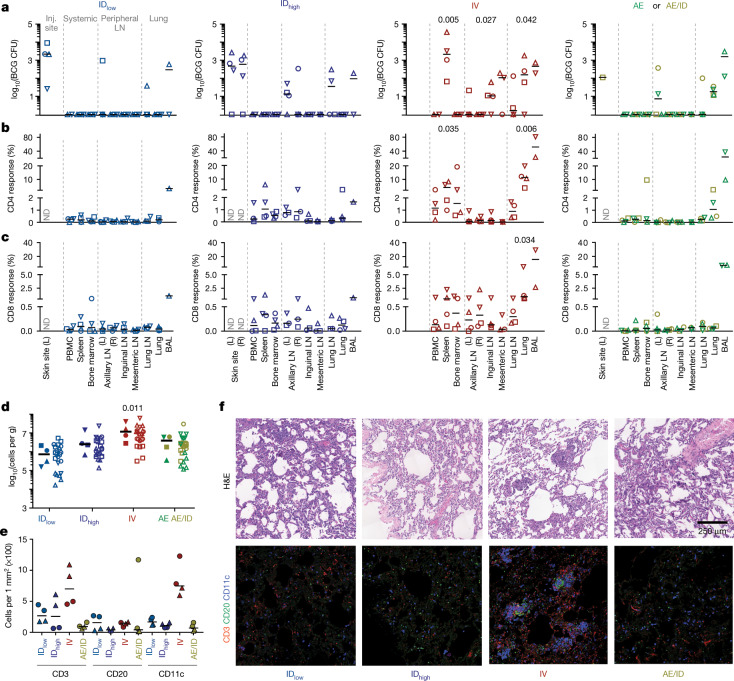
BCG CFUs and immune responses in tissues one month after BCG immunization. NHPs (cohorts 5a–c: ID_low_, ID_high_ and IV, *n* = 4 NHPs; AE and AE/ID, *n* = 2 NHPs) were euthanized one month after vaccination to quantify BCG and T cell responses in tissues. **a**, BCG CFUs at vaccination site(s) (skin, ID only) and in various tissues (per ml blood or bone marrow; per whole spleen, LN or lung lobe; or per total BAL collected). L, left; R, right; ND, not determined. **b**, **c**, Frequency of memory CD4 (**b**) and CD8 (**c**) T cells producing IFNγ, IL-2, TNF or IL-17 after PPD stimulation. Matched symbols within each vaccine group are the same macaque. Kruskal–Wallis tests were run and reported *P* values represent Dunn’s multiple comparison test comparing each group to the ID_low_ group. **d**, Total viable cells per gram of lung tissue for each vaccine regimen; data are shown as the median of four macaques per group (solid symbols, six lung lobes from each NHP are averaged) or as counts for each lung lobe (*n* = 24 lobes) from all NHPs (open symbols with lobes from same macaque matched). Kruskal–Wallis test was run on medians; Dunn’s adjusted *P* values are from comparing each group to the ID_low_ group. **e**, Quantification of CD3^+^, CD20^+^ and CD11c^+^ cells from two lung sections per NHP (matched symbols, *n* = 2 macaques). **f**, Representative (one out of four) 1 mm^2^ lung sections from each BCG regimen stained with haematoxylin and eosin (H&E; top) or with antibodies against CD3^+^ T cells (red), CD20^+^ B cells (green), and CD11c^+^ macrophages or dendritic cells (blue). Source Data

**Extended Data Fig. 10:**
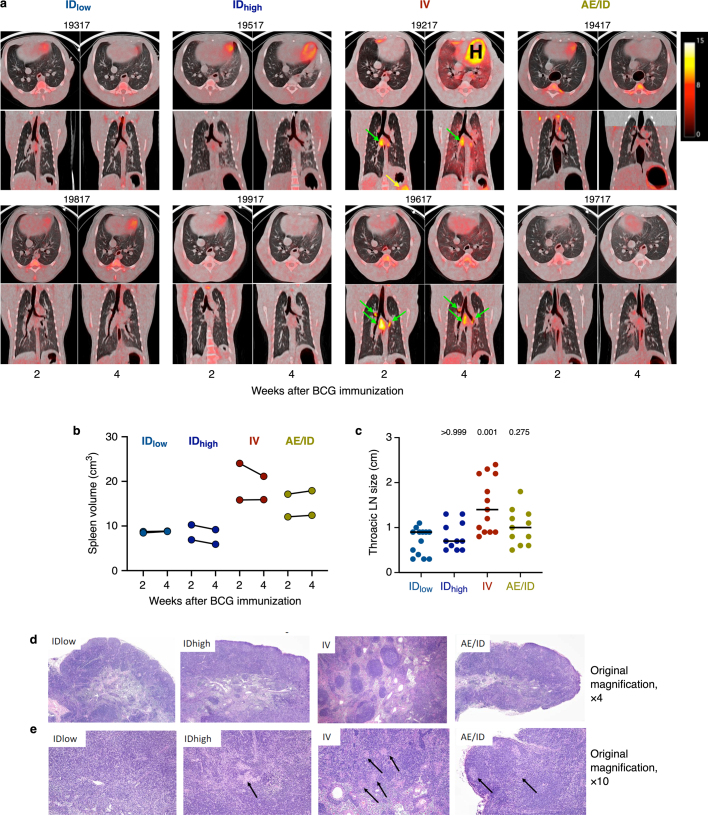
Inflammation and gross and histopathological assessment after BCG vaccination. **a**, Serial FDG PET–CT scans at 2 and 4 weeks after BCG vaccination showed increased metabolism (surrogate for inflammation) localized to the lung LNs (green arrows), lung lobes and spleen (yellow arrow) elicited by the IV but not by other routes (cohort 5a, b, *n* = 2 macaques). Warm colours indicate increased FDG retention; scale represents standardized uptake values. NHP ID numbers are listed above each scan; ‘H’ denotes the heart. **b**, Spleen volume was calculated from CT scans at 2 and 4 weeks after BCG vaccination (*n* = 2 macaques). At these time points, animals given IV BCG had approximately twofold larger spleens than those given ID BCG, with AE/ID BCG NHPs also displaying modestly enlarged spleens. **c**, Thoracic LNs were measured at necropsy, 4 weeks after BCG vaccination (*n* = 2 macaques); LNs from IV BCG NHPs were enlarged compared to those from ID_low_ NHPs. Kruskal–Wallis test was run; Dunn’s adjusted *P* values are reported comparing each vaccine group to the ID_low_ group. **d**, **e**, H&E-stained sections of thoracic LNs from vaccinated NHPs (*n* = 2 macaques), 4 weeks after BCG vaccination. **d**, General structure with respect to cortical and medullary architecture and appearance was normal in LNs from ID_low_, ID_high_ and AE/ID vaccinated NHPs. The thoracic LNs from the IV-vaccinated macaques demonstrated marked follicular lymphoid hyperplasia, with enlarged, prominent, variably sized follicles, often with active, expanded germinal centres. Original magnification, ×4. **e**, Small, non-necrotizing epithelioid histiocytic aggregates (non-necrotizing granulomas, black arrows) were abundantly disseminated within thoracic LNs from the IV BCG macaques. In the AE/ID NHPs, a wide nodal distribution of such lesions was also seen, although granuloma numbers and density were substantially less. The ID_high_ NHPs had only one observable granuloma in a single thoracic LN and in the ID_low_ NHPs, no such structures were evident. Original magnification, ×10. Source Data

To provide insight into the potential mechanisms of IV-BCG-induced protection, we quantified BCG CFUs and T cell responses in tissues 1 month after vaccination. BCG was detected at the skin site(s) of injection and draining axillary LNs in ID-BCG-vaccinated NHPs, but not in lung lobes (Fig. 3a). In AE- or AE/ID-BCG-vaccinated NHPs, BCG was detected primarily in lung lobes and BAL. By contrast, BCG was detected in the spleen of all four IV-BCG-vaccinated NHPs, as well as in BAL, lung lobe, and peripheral and lung LNs (Fig. 3a). Indeed, PET–CT scans at 2 and 4 weeks after BCG vaccination showed increased metabolism localized to lung LNs, lung lobes and spleen elicited by the IV but not by other routes (Extended Data Fig. 10a).

CD4 T cell responses in IV-BCG-immunized NHPs were increased in spleen and lung compared to ID_low_ NHPs (Fig. 3b), consistent with detection of BCG at the same sites. Moreover, CD4 T cell responses were observed in systemic sites such as PBMCs, bone marrow and peripheral LNs. CD8 responses were highest in lung lobes, BAL and spleen after IV BCG (Fig. 3c). After ID_high_ BCG vaccination, CD4 T cell responses were detected in spleen, bone marrow and axillary LNs, but were limited in lung lobes and lung LNs, whereas responses in AE groups were confined to the lung and BAL. Collectively, these data indicate compartmentalization of BCG detection and T cell immunity by vaccine route, which highlights the systemic distribution of immune responses after IV BCG versus the more limited and localized responses following ID and AE delivery.

Further analysis of lung tissue one month after vaccination showed increased cell counts (Fig. 3d) after IV BCG with increased numbers of CD3^+^ T cells and CD11c^+^ antigen-presenting cells (Fig. 3e). These clustered into ‘microgranulomas’ that were histologically distinct from bronchus-associated lymphoid tissue (BALT) (Fig. 3f). IV-BCG-vaccinated macaques had transient splenomegaly as well as enlarged thoracic LNs that contained non-necrotizing granulomas and lymphoid follicular hyperplasia, often with active germinal centres (Extended Data Fig. 10b–e).

**Extended Data Fig. 11:**
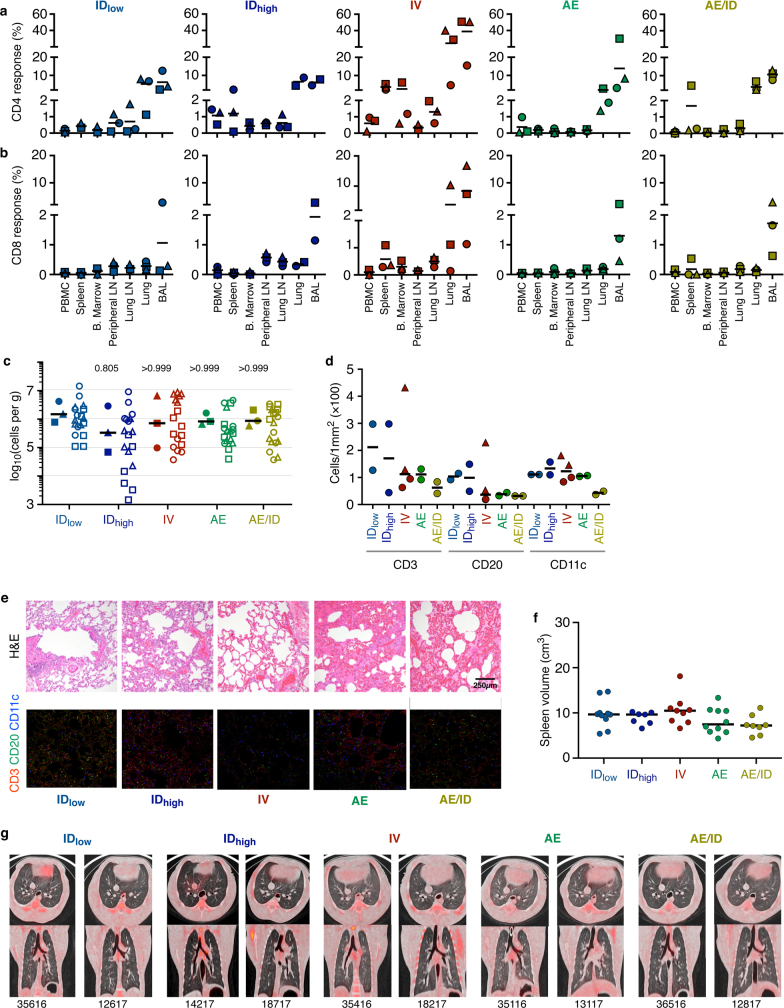
Immune response to BCG 6 months after vaccination. Analysis of tissue T cell responses, lung cell counts, immunohistochemistry, splenic volume and PET–CT scans was performed 6 months after BCG vaccination. **a**–**c**, A separate cohort (cohort 3, *n* = 3 macaques) was vaccinated with BCG in parallel to the challenge study with the purpose of assessing immune responses in various tissues 6 months after BCG (the time point at which macaques would be challenged). **a**, **b**, Frequency of memory CD4 (**a**) and CD8 (**b**) T cells producing any combination of IFNγ, IL-2, TNF, or IL-17 in response to Mtb WCL stimulation in the PBMC, spleen, bone marrow, peripheral LN, lung LN, lung tissue and BAL. Six months after IV BCG, immunized NHPs maintained increased frequencies of antigen-responsive T cells in spleen, BAL and lung lobes. Individual LN and lung lobe responses were averaged per macaque. Data points are individual macaques with symbols matched across tissues within a vaccine group; horizontal bar indicates the mean response. **c**, Number of cells recovered per gram of lung tissue for each NHP; the increased numbers of total cells observed at 1 month post-BCG (Fig. 3d) were not detected at 6 months post-BCG. Data are shown as the median of 3 macaques per group (solid symbols, counts from six lung lobes per animal are averaged) or as counts for individual lung lobes for each animal (open symbols; lobes from the same animal have matched symbols). Kruskal–Wallis test was used, and *P* values represent Dunn’s multiple comparison test comparing each vaccine group to the ID_low_ group. **d**, Quantification of CD3^+^, CD20^+^, CD11c^+^ cells from two lung sections (matched symbols) from 1–2 macaques per group using Cell Profiler. **e**, Representative 1-mm^2^ lung sections from 1–2 macaques per vaccine group were stained with H&E or with antibodies against CD3^+^ T cells (red), CD20^+^ B cells (green), and CD11c^+^ macrophages or dendritic cells (blue). Neither the increase in numbers of T cells and CD11c^+^ cells nor the histopathological changes in lung sections from IV-BCG-immunized macaques observed at 1 month (Fig. 3e, f) were detected 6 months after BCG vaccination. **f**, Spleen volume was calculated from CT scans of 44 NHPs (cohorts 1–3) just before Mtb challenge (6 months after BCG vaccination) and was not significantly different among vaccine routes (Kruskal–Wallis test, *P* = 0.1643). Dots represent individual animals. **g**, Axial (top) and coronal (bottom) PET–CT scans of two representative macaques (*n* = 8–10) from each vaccine group 6 months after BCG, before Mtb infection. Animal ID numbers are shown below each set of scans. No detectable lung inflammation (FDG uptake) was observed in macaques from any vaccine group. Source Data

Six months after BCG vaccination (time of challenge), NHPs that received IV BCG maintained increased frequencies of antigen-responsive T cells in spleen, lung and BAL (Extended Data Fig. 11a, b). Notably, the numbers of total, CD3^+^ or CD11c^+^ cells in lung tissue had normalized, and lung histopathology, spleen size and FDG uptake in IV-BCG-vaccinated macaques were indistinguishable from ID_low_ BCG macaques (Extended Data Fig. 11c–g). Although BCG burden was not measured in these NHPs, no BCG (or Mtb) CFUs were detected in six out of ten IV-BCG-immunized, challenged macaques at 9 months after BCG. Collectively, these data suggest that BCG is cleared between 1 and 9 months after IV vaccination.

## T cells in lung tissue after BCG

**Fig. 4:**
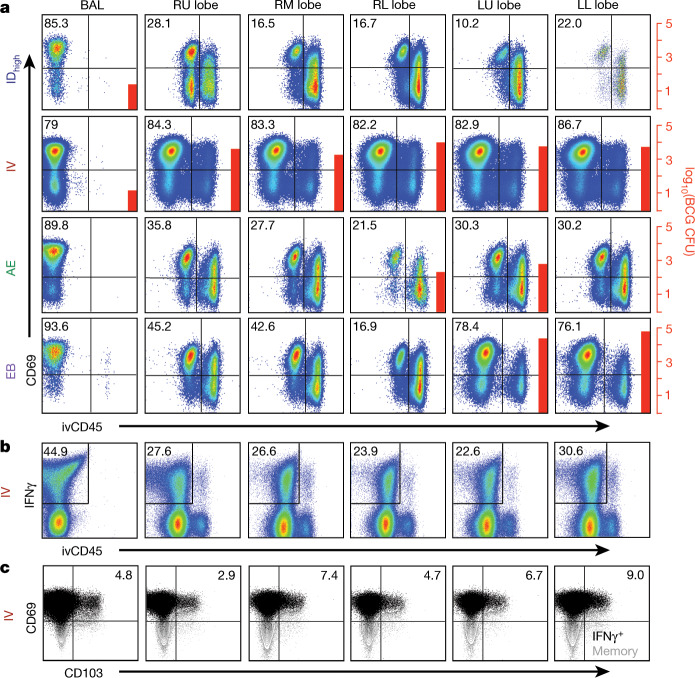
Detection of T cells in lung tissue after IV BCG immunization. **a**, One month after BCG vaccination, tissue-derived versus blood-derived cells in lung were delineated by injecting NHPs with a fluorochrome-conjugated anti-CD45 antibody (ivCD45) to label leukocytes in the vasculature. NHPs (cohort 6, *n* = 2 macaques) received 5 × 10^7^ CFUs BCG ID, IV, AE or endobronchially (EB) into the left lung. At necropsy, BCG CFUs were quantified in tissues and cells were stained immediately ex vivo for surface marker expression (**a**) or stimulated with Mtb whole-cell lysate (WCL) and stained for cytokine production (**b**, **c**). Plots show CD4 T cells from the BAL and lung lobes (RU, right upper; RM, right middle; RL, right lower; LU, left upper; LL, left lower) from one of two macaques per BCG regimen. **a**, Percentage of ivCD45^−^ (unstimulated) CD4 T cells expressing the tissue-resident/activation marker CD69; BCG CFUs (if detected) are indicated by red bars and right scale. **b**, Percentage of WCL-responsive (IFNγ^+^) CD4 T cells in BAL and lung tissue (ivCD45^−^) and (**c**) the percentage of IFNγ^+^ CD4 memory T cells expressing CD69 and CD103 after IV BCG vaccination. Source Data

To substantiate whether T cells isolated from lung lobes one month after IV BCG were T_RM_ cells, labelled anti-CD45 antibody was injected IV into NHPs just before necropsy—a technique shown to delineate tissue-derived (ivCD45^−^) from vasculature-derived (ivCD45^+^) leukocytes^29,30^. Ex vivo phenotypic analysis of CD69 expression (a marker of T_RM_ and/or T cell activation) in combination with ivCD45 staining revealed that more than 80% of CD4 T cells isolated from all lung lobes of IV-BCG-immunized NHPs were derived from the lung parenchyma (CD69^+^ivCD45^−^) (Fig. 4a). Of note, more than 1,000 BCG CFUs were cultured from every lung lobe in this macaque. By contrast, ID_high_ and AE BCG vaccination resulted in 16–35% tissue-derived (CD69^+^ivCD45^−^) CD4 T cells in the lung lobes, with few or undetectable BCG CFUs. T cells from BAL in all NHPs were uniformly CD69^+^ivCD45^−^. Similar results were observed in the CD8 T cell compartment of the same macaques (Supplementary Data 7).

**Extended Data Fig. 12:**
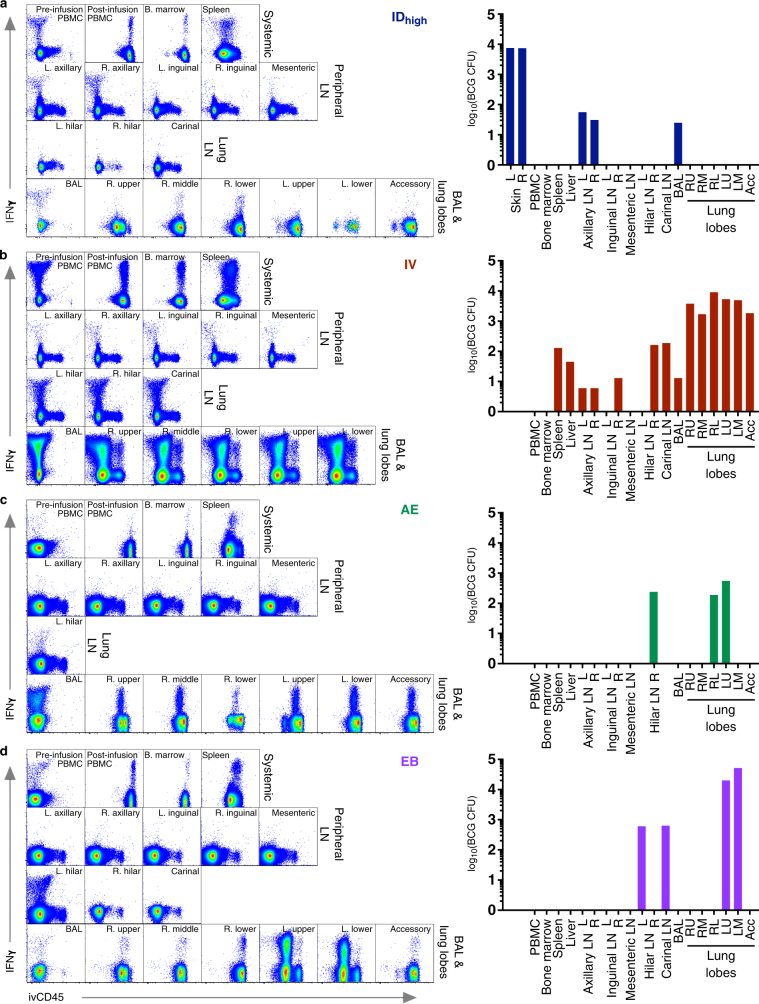
Determination of immune responses and BCG in tissues 1 month after immunization. NHPs (cohort 6, *n* = 2) were immunized with 5 × 10^7^ BCG CFUs (ID_high_ (**a**), IV (**b**), AE (**c**), or EB (**d**)); BCG CFUs and antigen-responsive T cells were measured in various tissues 1 month later. Before euthanasia, a fluorochrome-conjugated anti-CD45 antibody was injected intravenously (ivCD45) such that circulating (intravascular) leukocytes were uniformly stained (ivCD45^+^) while leukocytes in the tissue remained protected from staining (ivCD45^−^). To investigate whether antigen-responsive (IFNγ^+^) CD4 T cells were located in the ivCD45^−^ lung tissue compartment, cells isolated from lung lobes were re-stimulated in vitro with Mtb WCL and analysed by intracellular cytokine staining. FACS plots show memory CD4 T cells in all tissues collected from one of two NHPs per BCG regimen, organized by type/location (systemic, peripheral LN, lung LN, BAL and lung lobes). The BAL and lung responses from the IV BCG NHPs, shown in the bottom row of **b**, is reproduced from Fig. 4b. Pre-infusion PBMC indicates PBMCs isolated from whole blood collected just before anti-CD45 injection. Bar graphs show the number of BCG CFUs in each respective tissue for each animal (colour-coded by vaccine), if detected. Source Data

After in vitro antigen stimulation to assess antigen-responsive T cells in tissue, lung tissue-derived (ivCD45^−^) IFNγ-producing CD4 T cells were observed in all lung lobes and lung LNs of IV-BCG-immunized NHPs (Fig. 4b and Extended Data Fig. 12). Antigen-responsive lung T cells were largely CD69^+^ with a subset also expressing the tissue-homing marker CD103, which is expressed on some T_RM_ cells^31^ (Fig. 4c). Thus, these cells may represent bona fide T_RM_ cells, or recently activated T cells owing to the presence of BCG (Fig. 4a). Overall, these data show that IV BCG vaccination provided the highest level of protection concomitant with increased antigen-responsive T cells throughout lung tissue.

The increased detection of T cell responses in tissues containing BCG suggests that alternative approaches to lung vaccine delivery may be crucial for generating T_RM_ cells. Indeed, direct endobronchial instillation of BCG into a single lung lobe protected two out of eight NHPs against Mtb challenge in the same lobe^32^. To determine how endobronchial BCG would affect T cells in the lung parenchyma, BCG was instilled directly into the left lung lobes of NHPs. Approximately 75% of CD4 and CD8 T cells isolated from the two left lung lobes were CD69^+^ivCD45^−^, compared with 7–45% in the right lobes (Fig. 4a and Supplementary Data 7a). Notably, BCG CFUs (>10^4^) were detected in the left (but not right) lung lobes where the CD4 T cell response was highest (Extended Data Fig. 12). Collectively, these data suggest a general concordance between the presence of BCG in a given tissue after vaccination and the detection of antigen-responsive T cells.

## Immune associations of bacterial control

**Extended Data Fig. 13:**
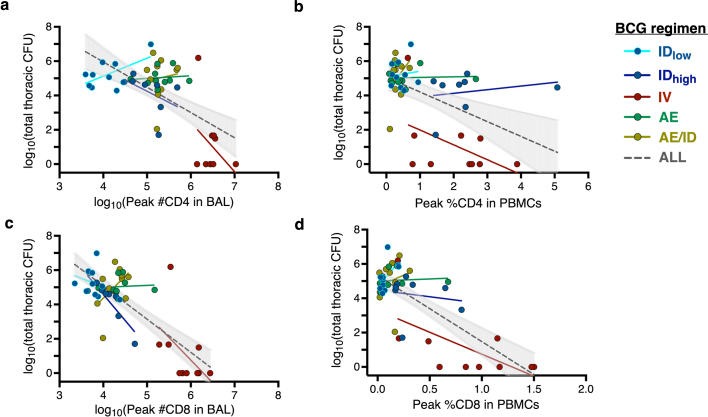
Relationships between peak T cell responses in BAL or PBMCs and total CFUs at necropsy. **a**–**d**, Linear regressions were used to test whether antigen-responsive CD4 (**a**, **b**) or CD8 (**c**, **d**) T cell numbers (BAL; **a**, **c**) or frequencies (PBMC; **b**, **d**) after BCG immunization are associated with disease severity (total CFUs). Results indicate that when controlling for all vaccine routes, peak CD4 T cells in the BAL and PBMC, and peak CD8 T cells in the BAL do not have a significant association with total CFUs (Supplementary Table 5a–c). Of note, in PBMCs, higher peak CD8 frequencies are associated with lower total CFUs after controlling for route (Supplementary Table 5d). Each dot represents an individual animal; coloured lines represent linear fit for each vaccine route. Dotted black lines represent linear fit for all vaccine routes combined (with 95% confidence interval shaded in grey). Source Data

Several multiple regressions were used to test whether peak antigen-responsive CD4 or CD8 T cells in the BAL or PBMCs after BCG immunization were associated with disease severity (Extended Data Fig. 13, Supplementary Tables 1 and 5). These analyses show that the route of BCG vaccination was the primary determinant of Mtb control with IV being the only regimen that afforded significant protection (Extended Data Fig. 8b).

## Discussion

The data demonstrating that IV BCG immunization results in markedly increased antigen-responsive T cells, including T cells systemically and throughout the lung parenchyma, and unprecedented protection against Mtb challenge, represent a major step forward in the field of TB vaccine research.

The concept of alternative immunization routes rather than the standard ID approach was suggested 50 years ago in NHP studies comparing IV and AE immunization^5–8^. More recently, decreased lung pathology and a trend towards increased survival was reported after IV BCG immunization compared with unvaccinated NHPs^33^. AE immunization with an attenuated Mtb strain enhanced cellular immunity in the BAL, and reduced lung pathology and bacterial burdens, after high-dose challenge 8 weeks later with a low virulence Mtb strain (CDC1551)^34^. In different method of pulmonary delivery, BCG instilled directly into the lower left lung lobe (that is, endobronchially), prevented infection and disease in two out of eight NHPs after repeated limiting-dose Mtb challenge in the same lung lobe, starting 13 weeks after vaccination^32^. The robust and localized T cell responses in lung tissue after direct BCG instillation (Fig. 4a and Extended Data Fig. 12d) provide a potential mechanistic difference between direct endobronchial and AE delivery that could influence protection. Finally, a cytomegalovirus (CMV) vector encoding Mtb antigens prevented TB disease in 14 out of 34 macaques across two studies, with 10 out of 14 being Mtb culture-negative^35^. In contrast to IV BCG immunization, all CMV-immunized macaques generated primary responses to Mtb antigens after challenge, suggesting that these vaccines elicit distinct mechanisms or kinetics of protection.

There are at least three immune mechanisms for how IV BCG may mediate protection. First, rapid elimination of Mtb may be due to the high magnitude of T cell responses in lung tissue. Our data are consistent with studies in mice that demonstrate the superior capacity of lung-localized T_RM_ cells to control TB disease^36,37^, and studies in NHPs showing that depletion of lung interstitial CD4 T cells during SIV infection of Mtb latently infected NHPs is associated with reactivation and dissemination^38^. Second, there is some evidence that antibodies can mediate control against Mtb in vivo or in vitro^39,40^. Antibody levels were higher in the BAL and plasma after IV BCG compared with other routes of vaccination, but declined to pre-vaccination levels in the BAL at the time of challenge (Extended Data Fig. 7). Third, IV BCG vaccination in mice induced epigenetically modified macrophages with enhanced capacity to protect against Mtb infection^41^, a process termed ‘trained immunity’^14,15^. Such an effect was dependent on BCG being detectable in the bone marrow; this was not observed one month after IV BCG vaccination in NHPs (Fig. 3a). Moreover, there was no increase in innate activation of PBMCs to non-Mtb antigens after IV BCG vaccination—a hallmark of trained immunity (Supplementary Data 3). Nonetheless, it is possible that any of these three mechanisms might act independently or together to mediate protection.

Because nine out of ten macaques were protected by IV BCG immunization (Fig. 2), we were unable to define an immune correlate of protection within this group (Extended Data Fig. 13); however, there were several unique quantitative and qualitative differences in the immune responses after IV BCG vaccination that may underlie protection. First, there were substantially higher numbers of Mtb antigen-responsive T cells in the BAL and PBMCs (Fig. 1b–e). Second, there was a unique CD4 T cell transcriptional profile in the BAL, which included upregulation of genes that have been associated with protection against TB (Fig. 1f–h). Third, and perhaps most noteworthy, was the large population of T cells in the tissue across all lung parenchyma lobes (Fig. 4, Extended Data Fig. 12 and Supplementary Data 7). Notably, although the BAL CD4 T cell responses were higher in ID_high_-, AE- and AE/ID-BCG-immunized NHPs compared to the ID_low_ BCG group, there was no increased protection. These data suggest that although measurement of BAL responses may provide greater insight into vaccine efficacy compared to blood, they may not fully reflect lung T_RM_ cell responses that might be the mechanism of protection.

In conclusion, this study provides a paradigm shift towards developing vaccines focused on preventing TB infection to prevent latency, active disease and transmission. The data support clinical development of IV delivery of BCG for use in adolescents or adults in whom modelling predicts the greatest effect on TB transmission^3^, and suggest that the IV route may improve the protective capacity of other vaccine platforms. This study also provides a benchmark against which future vaccines will be tested and a new framework to understand the immune correlates and mechanisms of protection against TB.

## Methods

### Macaques and sample size

Indian-origin rhesus macaques (*Macaca mulatta*) used in these studies are outlined in Extended Data Fig. 1c and Supplementary Table 1. All experimentation complied with ethical regulations at the respective institutions (Animal Care and Use Committees of the Vaccine Research Center, NIAID, NIH and of Bioqual, Inc., and of the Institutional Animal Care and Use Committee of the University of Pittsburgh). Macaques were housed and cared for in accordance with local, state, federal, and institute policies in facilities accredited by the American Association for Accreditation of Laboratory Animal Care (AAALAC), under standards established in the Animal Welfare Act and the Guide for the Care and Use of Laboratory Animals. Macaques were monitored for physical health, food consumption, body weight, temperature, complete blood counts, and serum chemistries. All infections were performed at the University of Pittsburgh where animals were housed in a biosafety level 3 facility.

The sample size for this study was determined using bacterial burden (measured as log_10_-transformed total thoracic CFUs) as the primary outcome variable. Initially, we planned to test BCG route efficacy by comparing IV, AE and AE/ID routes to ID_low_ vaccination and found that ten macaques per group would be sufficient to obtain over 90% power and adjusted the type I error rate for three group comparisons (*α* = 0.0167). After initiation of the first cohort of NHPs in this study, we elected to test the effect of dose on ID vaccination by adding an ID_high_ group (*n* = 8 macaques). The additional treatment group did not substantially reduce the power of the study. To detect a 1.5 difference in log_10_(total CFUs) with a pooled standard deviation of 0.8 (using previous data), we obtained over 90% (90.7%) power using 10 macaques per group with an adjusted type I error rate for 4 group comparisons (*α* = 0.0125). The comparison made between the ID_high_ (*n* = 8 macaques) and ID_low_ (*n* = 10 macaques) groups achieved 85.6% power detecting the same difference (log_10_(1.5)) and with an *α* = 0.0125.

### BCG vaccination

For Mtb challenge studies (cohorts 1–3), 3–5-year-old male (*n* = 32) and female (*n* = 20) rhesus macaques were randomized into experimental groups based on gender, weight and pre-vaccination CD4 T cell responses to PPD in BAL. Macaques were vaccinated at Bioqual, Inc. under sedation and in successive cohorts as outlined in Extended Data Fig. 1c. BCG Danish Strain 1331 (Statens Serum Institute, Copenhagen, Denmark) was expanded^42^, frozen at approximately 3 × 10^8^ CFUs ml^−1^ in single-use aliquots and stored at −80 °C. Immediately before injection, BCG (for all vaccine routes) was thawed and diluted in cold PBS containing 0.05% tyloxapol (Sigma-Aldrich) and 0.002% antifoam Y-30 (Sigma-Aldrich) to prevent clumping of BCG and foaming during aerosolization^43^. For ID vaccinations, BCG was injected in the left upper arm (5 × 10^5^ CFUs; ID_low_) or split across both upper arms (5 × 10^7^ CFUs; ID_high_) in a volume of 100–200 μl per site. IV BCG (5 × 10^7^ CFUs) was injected into the left saphenous vein in a volume of 2 ml; AE BCG (5 × 10^7^ CFUs) was delivered in a 2 ml volume via paediatric mask attached to a Pari eFlow nebulizer (PARI Pharma GmgH) that delivered 4 μM particles into the lung, as previously described^28^; AE/ID macaques were immunized simultaneously (5 × 10^7^ CFUs AE plus 5 × 10^5^ CFUs ID in left arm); EB BCG (5 × 10^7^ CFUs in 2 ml; cohort 6 only) was instilled into the left lung lobes using an endoscope. No loss of viability was observed for BCG after aerosolization. In pilot studies, lower doses of BCG were prepared and delivered as described above. Text refers to nominal BCG doses—actual BCG CFUs for vaccine regimens in every cohort were quantified immediately after vaccination and are reported in Extended Data Fig. 1c and Supplementary Table 1.

### Mtb challenge

Macaques (cohorts 1–3) were challenged by bronchoscope with 4–36 CFUs barcoded Mtb Erdman 6–10 months after BCG vaccination (Extended Data Fig. 1c and Supplementary Table 1) in a 2 ml volume as previously described^44^. Infectious doses across this range result in similar levels of TB disease in unvaccinated rhesus in this and previous studies^28^ (Supplementary Data 12). Clinical monitoring included regular monitoring of appetite, behaviour and activity, weight, erythrocyte sedimentation rate, Mtb growth from gastric aspirate and coughing. These signs, as well as PET–CT characteristics, were used as criteria in determining whether a macaque met the humane end point before the pre-determined study end point.

### PET–CT scans and analysis

PET–CT scans were performed using a microPET Focus 220 preclinical PET scanner (Siemens Molecular Solutions) and a clinical eight-slice helical CT scanner (NeuroLogica Corporation) as previously described^27,45–47^. 2-deoxy-2-(^18^F)fluorodeoxyglucose (FDG) was used as the PET probe. Serial scans were performed before, 4 and 8 weeks after Mtb, and before necropsy (cohorts 1–3) or at 2 and 4 weeks after BCG (cohorts 5a, b). OsiriX MD (v.10.0.1), a DICOM (Digital Imaging and Communications in Medicine) image viewer, was used for scan analyses, as described^47^. Lung inflammation was measured as total FDG activity within the lungs. A region of interest (ROI) was segmented which encompassed all lung tissue on CT and was then transferred to the co-registered PET scan. On the PET scan, all image voxels of FDG-avid pathology (Standard Uptake Value >2.3) were isolated and summated resulting in a cumulative standardized uptake value. To account for basal metabolic FDG uptake, total FDG activity was normalized to resting muscle resulting in a total lung inflammation value. Individual granulomas were counted on each CT scan. If granulomas were too small and numerous within a specific area to count individually or if they consolidated, quantification was considered to be too numerous to count. To measure the volume of the spleen, an ROI was drawn outlining the entire organ on each of the axial slices of the CT scan and the volume was computed across these ROIs (using a tool in OsiriX). Any scans for which visibility of the entire spleen was limited (*n* = 2 macaques) were excluded from this analysis.

### Necropsy, pathology scoring and Mtb and BCG burden

For challenge studies (cohorts 1–3), NHPs were euthanized 11–15 weeks after Mtb or at humane endpoint by sodium pentobarbital injection, followed by gross examination for pathology. A published scoring system^27^ was used to determine total pathology from each lung lobe (number and size of lesions), LN (size and extent of necrosis), and extrapulmonary compartments (number and size of lesions). All granulomas and other lung pathologies, all thoracic LNs, and peripheral LNs were matched to the final PET–CT scan and collected for quantification of Mtb. Each lesion (including granulomas, consolidations and clusters of granulomas) in the lung, all thoracic LNs, random sampling (50%) of each of the 7 lung lobes, 3–5 granulomas (if present) or random samples (30%) of spleen and liver, and any additional pathologies were processed to comprehensively quantify bacterial burdens. Suspensions were plated on 7H11 agar (Difco) and incubated at 37 °C with 5% CO_2_ for 3 weeks for CFU enumeration or formalin-fixed and paraffin-embedded for histological examination. CFUs were counted and summed to calculate the total thoracic bacterial burden for the macaque^17,27,48^. Mtb CFUs for every challenged macaque are listed in Supplementary Table 1.

To determine BCG CFUs, BAL, bone marrow aspirates, and blood were collected from NHPs before euthanasia. Individual lung lobes and thoracic and peripheral LNs, spleen, liver, and the skin site(s) of injection (if applicable) were excised. 0.5 ml of blood and bone marrow and 10% of retrieved BAL wash fluid were plated; approximately 1 g of tissue (or one whole LN or skin biopsy) was processed in water in gentleMACS M Tubes (Miltenyi Biotec) using a gentleMACS Dissociator (Miltenyi Biotec). Samples were plated and counted as above. Data are reported as CFUs ml^−1^ of blood or bone marrow, CFUs per total BAL collected, CFUs per one LN or skin biopsy, CFUs per lung lobe or spleen. CFUs from individual lung lobes and LNs of the same category (for example, hilar) were averaged for each NHP.

### Rhesus blood, BAL and tissue processing

Blood PBMCs were isolated using Ficoll-Paque PLUS gradient separation (GE Healthcare Biosciences) and standard procedures; BAL wash fluid (3 × 20 ml washes of PBS) was centrifuged and cells were combined before counting, as described^28^. LNs were mechanically disrupted and filtered through a 70-μm cell strainer. Lung and spleen tissues were processed using gentleMACS C Tubes and Dissociator in RPMI 1640 (ThermoFisher Scientific). Spleen mononuclear cells were further separated using Ficoll-Paque. Lung tissue was digested using collagenase, Type I (ThermoFisher Scientific) and DNase (Sigma-Aldrich) for 30–45 min at 37 °C with shaking, followed by passing through a cell strainer. Single-cell suspensions were resuspended in warm R10 (RPMI 1640 with 2 mM l-glutamine, 100 U ml^−1^ penicillin, 100 μg ml^−1^ streptomycin, and 10% heat-inactivated FBS; Atlantic Biologicals) or cryopreserved in FBS containing 10% DMSO in liquid nitrogen.

### Multiparameter flow cytometry

Generally, longitudinal PBMC samples were batch-analysed for antigen-specific T cell responses or cellular composition at the end of the study from cryopreserved samples whereas BAL and tissue (necropsy) samples were analysed fresh. Cryopreserved PBMC were washed, thawed and rested overnight in R10 before stimulation, as described^28^. For T cell stimulation assays, 1–5 million viable cells were plated in 96-well V-bottom plates (Corning) in R10 and incubated with R10 alone (background), or with 20 μg ml^−1^ tuberculin PPD (Statens Serum Institut, Copenhagen, Denmark), 20 μg ml^−1^ H37Rv Mtb WCL (BEI Resources), or 1 μg ml^−1^ each of ESAT-6 and CFP-10 peptide pools (provided by Aeras, Rockville, MD) for 2 h before adding 10 μg ml^−1^ BD GolgiPlug (BD Biosciences). The concentrations of PPD and WCL were optimized to detect CD4 T cell responses; however, protein antigen stimulation may underestimate CD8 T cell responses. For logistical reasons, cells were stimulated overnight (14 h total) before intracellular cytokine staining. For cellular composition determination, cells were stained immediately ex vivo after processing or after thawing. Antibody and tetramer information for each flow cytometry panel is listed in Supplementary Data 8–11. Generally, cells were stained as follows (not all steps apply to all panels, all are at room temperature): Washed twice with PBS/BSA (0.1%); 20-min incubation with rhesus MR1 tetramer^49^ (NIH Tetramer Core Facility) in PBS/BSA; washed twice with PBS; live/dead stain in PBS for 20 min; washed twice with PBS/BSA; 10-min incubation with human FcR blocking reagent (Miltenyi Biotec); incubation with surface marker antibody cocktail in PBS/BSA containing 1× Brilliant Stain Buffer Plus (BD Biosciences) for 20 min; washed three times with PBS/BSA (0.1%); 20 min incubation BD Cytofix/Cytoperm Solution (BD Biosciences); washed twice with Perm/Wash Buffer (BD Biosciences); 30 min incubation with intracellular antibody cocktail in Perm/Wash Buffer containing 1× Brilliant Stain Buffer Plus; washed thrice with Perm/Wash Buffer. For Ki-67 staining, samples were stained for surface markers and cytokines as described above, followed by nuclear permeabilization using eBioscience Foxp3/Transcription Factor Staining Buffer (ThermoFisher Scientific) and incubation with antibody against Ki-67 following kit instructions. Data were acquired on either a modified BD LSR II or modified BD FACSymphony and analysed using FlowJo software (v.9.9.6 BD Biosciences). Gating strategies can be found in Supplementary Data 8–11. All cytokine data presented graphically are background-subtracted.

### Intravascular CD45 staining

One month after BCG vaccination, macaques in each cohort 6 (*n* = 2 macaques per group) received an IV injection of Alexa Fluor 647-conjugated anti-CD45 antibody (ivCD45; 60 μg kg^−1^, clone MB4-6D6, Miltenyi Biotec) 5 min before euthanasia. Blood was collected before anti-CD45 injection as a negative control, and before euthanasia as a positive control. NHPs underwent whole body perfusion with cold saline before tissue collection. Tissues were processed for BCG CFU quantification and flow cytometric analysis as described above. Staining panels used were as in Supplementary Data 9, with the omission of the APC-conjugated antibodies.

### Immunohistochemistry

Embedded tissue sections were deparaffinized (100% xylenes, 10 min; 100% ethanol, 5 min; 70% ethanol, 5 min), boiled under pressure for 6 min in antigen retrieval buffer (1× Tris EDTA, pH 9.0), and cooled. Sections were blocked in PBS (1% BSA) in a humidified chamber at room temperature for 30 min followed by staining for CD3 (CD3-12, Abcam), CD11c (5D11, Leica), and CD20 (Thermo Scientific, RB-9013-PO) for 18 h at 4 °C in a humidified chamber. After washing with PBS in coplin jars, sections were incubated for 1 h at room temperature with conjugated anti-rabbit IgG Alexa Fluor 488 (Life Technologies, A21206), anti-rat IgG Alexa Fluor 546 (Invitrogen, A11081), and anti-mouse IgG Alexa Fluor 647 (Jackson ImmunoResearch, 7 5606-150). After washing, coverslips were applied using Prolong Gold anti-fade with Dapi mounting media (Life Technologies). Slides were cured for 18–24 h before imaging on an Olympus FluoView FV1000 confocal microscope. Lung sections were imaged and two random representative 1 mm^2^ ROIs from each macaque were analysed using CellProfilerv2.2.0. Pipelines were designed for analysis by adding modules for individual channel quantification based on pixel intensity and pixel size providing a numerical value for each cell type and total cells. Histological analyses were performed by a veterinary pathologist (E.K.) in a blinded fashion on H&E-stained sections from all tissues obtained.

### ELISpot and Luminex

IFNγ ELISpots were performed at 0, 4, 6 and 8 weeks after Mtb and at necropsy. One day before use, hydrophobic high protein binding membranes 96-well plates (Millipore Sigma) were hydrated with 40% ethanol, washed with sterile water, and coated with anti-human/monkey IFNγ antibody (15 μg ml^−1^, MT126L, MabTech) overnight at 4 °C. Plates were washed with HBSS and blocked with RPMI with 10% human AB serum for 2 h at 37 °C with 5% CO_2_. Approximately 200,000 PBMCs per well were incubated in RPMI supplemented with l-glutamate, HEPES and 10% human AB serum containing 2 μg ml^−1^ ESAT-6 or CFP-10 peptide pools for 40–48 h at 37 °C with 5% CO_2_. Medium alone or phorbol 12,13-dubutyrate (12.5 μg ml^−1^) plus ionomycin (37.5 μg ml^−1^) were added as negative (background) and positive controls, respectively. To develop, plates were washed with PBS and biotinylated anti-human IFNγ antibody (2.5 μg ml^−1^, 7-B6-1, MabTech) was added for 2 h at 37 °C with 5% CO_2_. After washing, streptavidin-horseradish peroxidase (1:100, MabTech) was added for 45 min at 37 °C with 5% CO_2_. Spots were stained using AEC peroxidase (Vector Laboratories, Inc.) per the manufacturer’s instructions and counted manually on an ELISpot plate reader. Data are reported as average ELISpots from duplicate background-subtracted wells. Wells with confluent spots were described as too numerous to count.

To measure innate cytokine production following BCG immunization, cryopreserved PBMC were batch-analysed. Cells were thawed and resuspended in warm R10. Then, 5 × 10^5^ cells per well in 96-well V-bottom plates were rested overnight at 37 °C with 5% CO_2_. Cells were resuspended in Trained Immunity Media^15^ plus H37Rv Mtb whole cell lysate (BEI Resources, 20 μg ml^−1^), heat-killed *Staphylococcus aureus* (InvivoGen, 1 × 10^6^ per ml), *Escherichia coli* LPS (Sigma-Aldrich, 1 ng ml^−1^), or RPMI and incubated for 24 h at 37 °C with 5% CO_2_ before collecting supernatants. Cytokine and chemokine measurements were determined using a MILLIPLEX NHP cytokine multiplex kit per instructions (Millipore Sigma) and analysed on a Bio-Plex Magpix Multiplex Reader (Bio-Rad).

### Antibody ELISAs

IgG, IgA and IgM titres to Mtb H37Rv WCL were assessed in plasma and tenfold concentrated BAL fluid. WCL was used based on greater sensitivity compared to PPD, culture filtrate protein, or lipoarabinomannan. 96-well MaxiSorp ELISA plates (Nunc) were coated overnight at 4 °C with 0.1 μg of WCL. Plates were blocked with PBS/FBS (10%) for 2 h at room temperature and washed with PBS/TWEEN 20 (0.05%). 1:5 serially diluted plasma or concentrated BAL fluid (8 dilutions per sample) was incubated at 37 °C for 2 h, followed by washing. Then, 100 μl of goat anti-monkey HRP-conjugated IgG h+l (50 ng ml^−1^; Bethyl Laboratories, Inc.), IgA α chain (0.1 μg ml^−1^, Rockland Immunochemicals Inc.), or IgM α chain (0.4 μg ml^−1^, Sera Care) was added for 2 h at room temperature, followed by washing. Ultra TMB substrate (100 μl; Invitrogen) was added for 12 min followed by 100 μl 2 N sulfuric acid. Data were collected on a Spectramax i3X microplate reader (Molecular Devices) at 450 nm using Softmax Pro and presented either as endpoint titer (reciprocal of last dilution with an OD above the limit of detection or 2× the OD of an empty well) at 0.2 for IgG and IgA, or midpoint titer for IgM where samples did not titre to a cut off of 0.2.

### Single-cell transcriptional profiling

High-throughput single-cell mRNA sequencing by Seq-Well was performed on single-cell suspensions obtained from NHP BAL, as previously described^24^. Approximately 15,000 viable cells per sample were applied directly to the surface of a Seq-Well device. At each time point after BCG, two arrays were run for each sample—one unstimulated and one stimulated overnight with 20 μg ml^−1^ of PPD in R10.

#### Sequencing and alignment

Sequencing for all samples was performed on an Illumina Nova-Seq. Reads were aligned to the *M. mulatta* genome using STAR^50^, and the aligned reads were then collapsed by cell barcode and unique molecular identifier (UMI) sequences using DropSeq Tools v.1 to generate digital gene expression (DGE) matrices, as previously described^24,51^. To account for potential index swapping, we merged all cell barcodes from the same sequencing run that were within a hamming distance of 1.

#### Analysis of single-cell sequencing data

For each array, we assessed the quality of constructed libraries by examining the distribution of reads, genes and transcripts per cell. For each time point, we next performed dimensionality reduction (PCA) and clustering as previously described^52,53^. We visualized our results in a two-dimensional space using UMAP^54^, and annotated each cluster based on the identity of highly expressed genes. To further characterize substructure within cell types (for example, T cells), we performed dimensionality reduction (PCA) and clustering over those cells alone as previously described^24^. We then visualized our results in two-dimensional space using *t*-distributed stochastic neighbour embedding (*t*-SNE)^24^. Clusters were further annotated (that is, as CD4 and CD8 T cells) by cross-referencing cluster-defining genes with curated gene lists and online databases (that is, SaVanT andGSEA/MsigDB)^55–57^.

#### Module identification

Data from stimulated or unstimulated T cells at week 13 or 25 was subset on significant principal components as previously described^24^ and, for those principal components, on genes with significant loadings as determined through a randomization approach (‘JackStraw’)^52^. These matrices were then used as the inputs for WGCNA^58^. Following the WGCNA tutorial (https://horvath.genetics.ucla.edu/html/CoexpressionNetwork/Rpackages/WGCNA/Tutorials/), we chose an appropriate soft power threshold to calculate the adjacency matrix. As scRNA-seq data is affected by transcript drop-out (failed capture events), adjacency matrices with high power further inflate the effect of this technical limitation, and yield few correlated modules. Therefore, when possible, we chose a power as suggested by the authors of WGCNA (that is, the first power with a scale free topology above 0.8); however, if this power yielded few modules (fewer than three), we decreased our power. We then generated an adjacency matrix using the selected soft power and transformed it into a topological overlap matrix (TOM). Subsequently, we hierarchically clustered this TOM, and used the cutreeDynamic function with method ‘tree’ to identify modules of correlated genes using a dissimilarity threshold of 0.5 (that is, a correlation of 0.5). To test the significance of the correlations observed in each module, we implemented a permutation test. Binning the genes in the true module by average gene expression (number of bins = 10), we randomly picked genes with the same distribution of average expression from the total list of genes used for module discovery 10,000 times. For each of these random modules, we performed a one-sided Mann–Whitney *U*-test between the distribution of dissimilarity values among the genes in the true module and the distribution among the genes in the random module. Correcting the resulting *P* values for multiple hypothesis testing by Benjamini–Hochberg false discovery rate correction, we considered the module significant if fewer than 500 tests (*P* < 0.05) had false discovery rate > 0.05.

#### Gene module enrichments

To characterize the seven significant gene modules identified among in vitro-stimulated T cells collected 13 weeks after vaccination, we performed an enrichment analysis using databases of gene expression signatures (SaVanT and GSEA/MsigDb). Specifically, the enrichments in the Savant database, which includes signatures from ImmGen, mouse body atlas and other datasets (http://newpathways.mcdb.ucla.edu/savant-dev/), were performed using genes included in significant modules with a background expression set of 32,681 genes detected across single cells using Piano (https://varemo.github.io/piano/).

### Statistical methods

All reported *P* values are from two-sided comparisons. For continuous variables, vaccine routes were compared using a Kruskal–Wallis test with Dunn’s multiple comparison adjustment or one-way ANOVA with Dunnett’s multiple comparison adjustment (comparing all routes to ID_low_ BCG). Fisher’s exact tests were run for multiple CFU thresholds (evaluating protection) to assess the association between vaccine route and protection from Mtb (Extended Data Fig. 8b). A permutation test^59^ was used to compare fractional distributions (pie charts) of all vaccine groups to ID_low_ BCG. For clinical parameters, combined pre-vaccination measurements from all NHPs were compared against distributions from every vaccine group at every time point using Dunnett’s test for multiple comparisons. To assess whether post-vaccination antigen-responsive CD4 or CD8 T cells in the BAL or PBMCs are associated with disease severity, we first calculated peak T cell responses for each NHP over the course of vaccine regimen. The log_10_-transformed CD4 and CD8 cell counts were calculated within BAL and frequencies of CD4 and CD8 cells were calculated within PBMCs. To assess the effects of vaccine route and T cells on log_10_-transformed total CFUs, several multiple linear regressions were run in JMP Pro (v.12.1.0). Peak T cell responses and CFUs for each macaque included in these analyses are provided in Supplementary Table 1; detailed regression output (including model fit, ANOVA results, effect tests and parameter estimates) is provided in Supplementary Table 5. Cytokine production for trained immunity assay was compared using a two-way ANOVA and Dunnett’s multiple comparison test. Serial PBMC responses to CFP, ESAT-6 or CFP-10 by IFNγ ELISpot were analysed by using a Wilcoxon signed-rank test to compare pre-infection versus 12 weeks post-infection time points (within each vaccine route).

### Reporting summary

Further information on research design is available in the Nature Research Reporting Summary linked to this paper.

## Online content

Any methods, additional references, Nature Research reporting summaries, source data, extended data, supplementary information, acknowledgements, peer review information; details of author contributions and competing interests; and statements of data and code availability are available at 10.1038/s41586-019-1817-8.

## Supplementary information

Supplementary Data This file contains Supplementary Data files 1-12.

Reporting Summary

Supplementary Table Supplementary Table 1: T cell responses and challenge outcome for each Mtb-challenged NHP. Listed for each animal challenged with Mtb: BCG route, vaccination date, BCG dose; NIH and Pittsburgh animal identities and challenge cohort; peak CD4 and CD8 T cell responses in BAL (log_10_ number) and PBMC (%); Mtb infection date and dose, necropsy date, number of granulomas (grans) seen on CT and total lung FDG activity on scan immediately preceding necropsy, gross pathology score, extrapulmonary (EP) score, total thoracic CFU (with and without log_10_ transformation), total CFU within the lungs, and total CFU within the LN. tntc: too numerous to count.

Supplementary Table Supplementary Table 2: Stimulation-inducible gene modules. Statistically significant modules of correlated gene expression.

Supplementary Table Supplementary Table 3: Savant and GSEA/MsigDb gene enrichments. Gene enrichments of the 7 significant modules in databases of gene expression signatures.

Supplementary Table Supplementary Table 4: Module 2 differential expression results. Differentially expressed genes between module 2-positive and negative T cells.

Supplementary Table Supplementary Table 5: Regression results for T cell responses on total CFU. Several multiple regressions were used to test whether CD4 or CD8 T cell numbers (BAL) or frequencies (PBMC) after BCG immunization are associated with disease severity (Extended Data Fig. 13). Results indicate that vaccine route has a significant effect on total CFU, controlling for peak CD4 and CD8 T cells in the BAL and peripheral blood. Peak CD4 counts and frequencies in BAL and PBMCs, respectively, are not significantly correlated with total CFU when controlling for vaccine route (Supplementary Tables 5a–c). In PBMC, higher peak CD8 frequencies are associated with lower total CFU when controlling for route (Supplementary Table 5d). Under the expanded estimates sections, the t-tests are testing if each term differs significantly from the overall mean. Note that for all four models, IV route total CFU is significantly lower (negative estimate terms) than the overall total CFU.

## Source data

Source Data Fig. 1

Source Data Fig. 2

Source Data Fig. 3

Source Data Fig. 4

Source Data Extended Data Fig. 2

Source Data Extended Data Fig. 3

Source Data Extended Data Fig. 4

Source Data Extended Data Fig. 5

Source Data Extended Data Fig. 6

Source Data Extended Data Fig. 7

Source Data Extended Data Fig. 8

Source Data Extended Data Fig. 9

Source Data Extended Data Fig. 10

Source Data Extended Data Fig. 11

Source Data Extended Data Fig. 12

Source Data Extended Data Fig. 13

## Data Availability

All relevant data are available from the corresponding author upon reasonable request. Supplementary Table 1 provides peak immune data and post-challenge data for individual NHPs and Supplementary Table 5 provides regression analyses that support Extended Data Fig. 13. Supplementary Tables 2–4 include stimulation-inducible module genes, gene enrichments for modules, and differentially expressed genes that support transcriptional profiling data. RNA-sequencing data that support this study have been deposited in the Gene Expression Omnibus (GEO) under accession number GSE139598. Source Data for Figs. 1–4 and Extended Data Figs. 2–13 are provided with the paper.

## Code availability

All R code used for analysis of Seq-Well data is available upon request.
