# Supplementary material for: Prevention of tuberculosis in macaques after intravenous BCG immunization
Source: Nature. 2020 Jan 1;577(7788):95–102. doi: 10.1038/s41586-019-1817-8 (PMC7015856; doi:10.1038/s41586-019-1817-8)
Supplement: Supplementary file 1 — This file contains Supplementary Data files 1-12. [file 41586_2019_1817_MOESM1_ESM.pdf]

nature

https://doi.org/10.1038/s41586-019-1817-8

Supplementary information

Prevention of tuberculosis in macaques after intravenous BCG immunization

In the format provided by the authors and unedited

Patricia A. Darrah, Joseph J. Zeppa, Pauline Maiello, Joshua A. Hackney, Marc H. Wadsworth II, Travis K. Hughes, Supriya Pokkali, Phillip A. Swanson II, Nicole L. Grant, Mark A. Rodgers, Megha Kamath, Chelsea M. Causgrove, Dominick J. Laddy, Aurelio Bonavia, Danilo Casimiro, Philana Ling Lin, Edwin Klein, Alexander G. White, Charles A. Scanga, Alex K. Shalek, Mario Roederer, JoAnne L. Flynn & Robert A. Seder

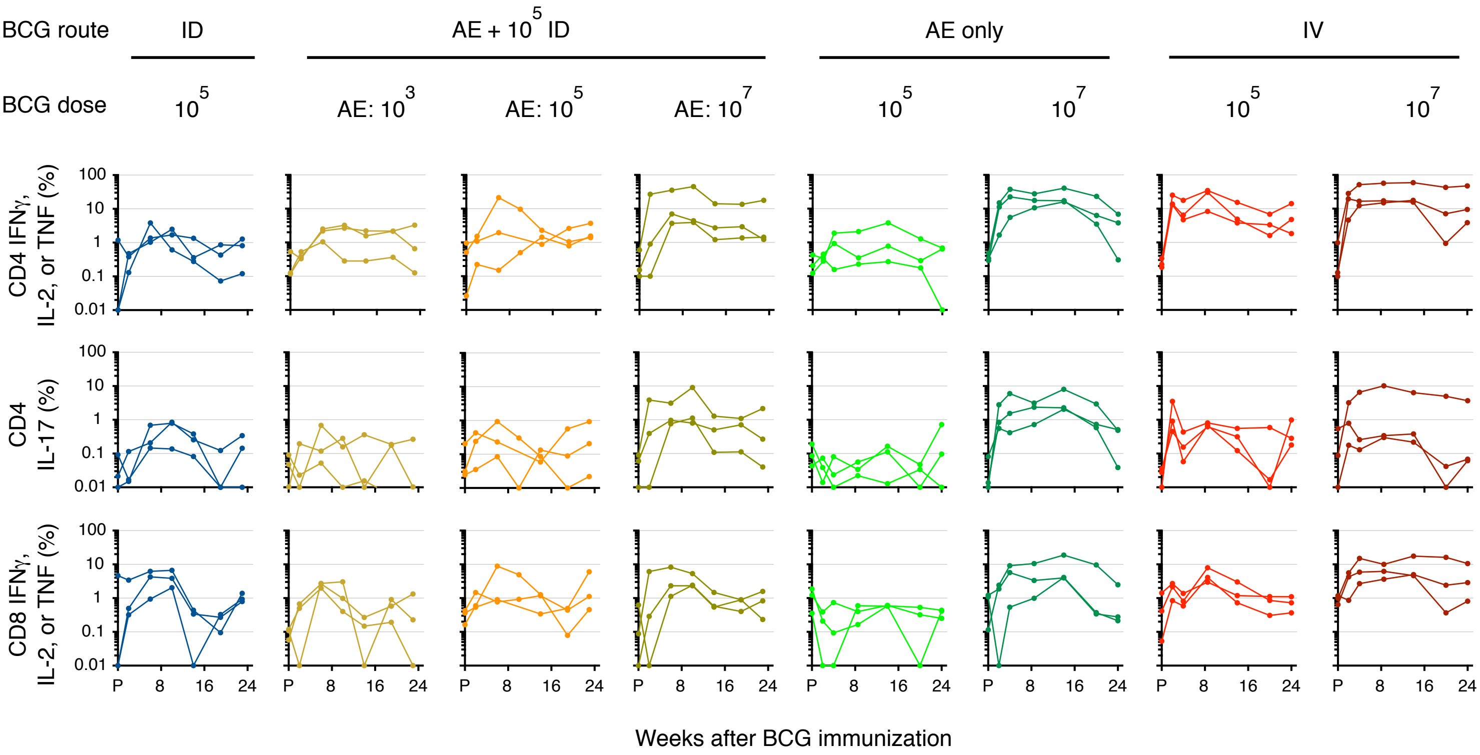

Supplementary Data 1: Dose-finding pilot study in NHP. To determine a BCG dose and vaccination route that would elicit greater immune responses in the BAL compared to the standard human BCG vaccine regimen (5×10^5 CFU ID), 3 NHP per group (24 total macaques, 22 male and 2 female; 6–9 years old) were immunized with BCG by different routes and nominal doses as follows: ID only (1×10^5 CFU); AE (1×10^3, 1×10^5, or 1×10^7 CFU) plus ID (1×10^5 CFU); AE only (5×10^5 or 5×10^7); IV only (5×10^5 or 5×10^7 CFU). Actual BCG doses can be found in **Extended Data Fig. 1c** (Pilot cohorts a–c). Prior to (Pre, P) and at various weeks after BCG immunization, BAL was collected from each macaque to perform *in vitro* T cell stimulation and flow cytometric cytokine analysis. Shown are the frequencies (log scale) of memory CD4 T cells producing any combination of IFNγ, IL-2, or TNF (top), memory CD4 T cells producing IL-17 (with or without other cytokines; middle), and memory CD8 T cells producing IFNγ, IL-2, or TNF (bottom) in response to PPD stimulation for 3 individual macaques through 24 weeks after BCG. No statistical comparisons were performed in this pilot study. For challenge studies, a dose of ~5×10^7 CFU BCG was selected based on the capacity of this dose to elicit robust cellular immunity when administered AE and IV.

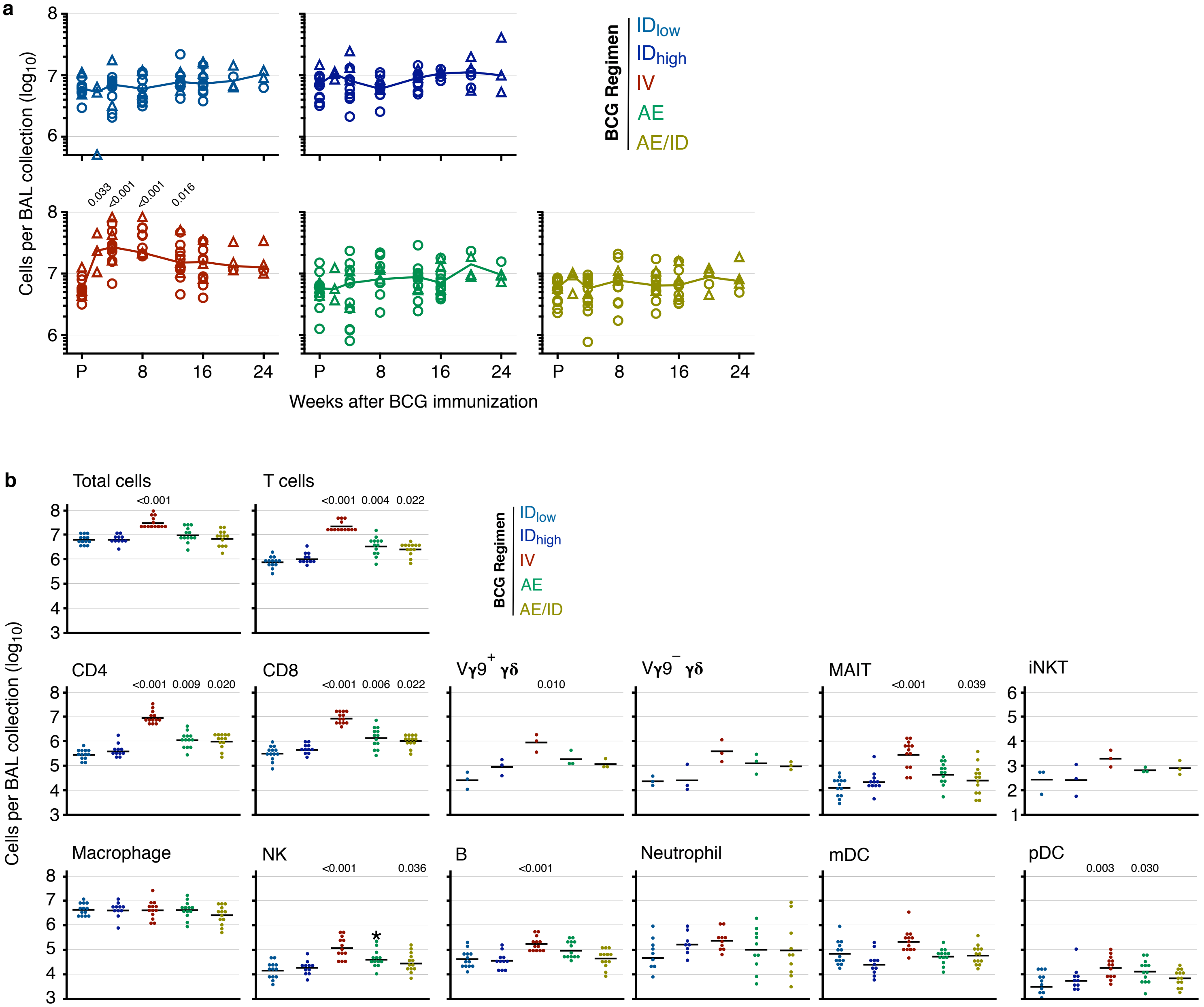

Supplementary Data 2: Cell counts in the BAL after BCG vaccination. **a**, Total number of viable, nucleated cells isolated from the BAL collected from BCG-immunized NHP (cohorts 1–4, n = 11–13 macaques) before vaccination (P) and after BCG, as determined by ethidium bromide/acridine dual-fluorescence automated counting. Circles represent individual macaques from challenge cohorts (1–3, n = 8–11) that were not lavaged within 8 week before challenge (per protocol); triangles are macaques from cohort 4 (n = 3) that were used for immunological measurements only and sampled through week 24 after BCG. Line represents the median for each group over time. **b**, Number of cells of indicated leukocyte population in the BAL of immunized NHP 8 weeks after BCG, as determined by flow cytometry (identified as in **Supplementary Data 8**). For each subset, the frequency of live cells was multiplied by the number of viable nucleated cells harvested from the BAL. Data points are individual macaques (n = 11–13 macaques except γδ, and iNKT cells, n = 3 macaques) and horizontal lines represent the geometric means that were used to generate **Fig. 1a**. T, T cells; B, B cells; mDC, myeloid dendritic cells; pDC, plasmacytoid dendritic cells; Neut, neutrophils; NK, NK cells; CD4, CD4 T cells; CD8, CD8 T cells; Vγ9+, Vγ9+ γδ T cells; MAIT, mucosal associated invariant T cells; iNKT, invariant natural killer T cells. Kruskal-Wallis tests were run and reported *P* values represent Dunn's multiple comparison test comparing each vaccine group to ID_{low}.

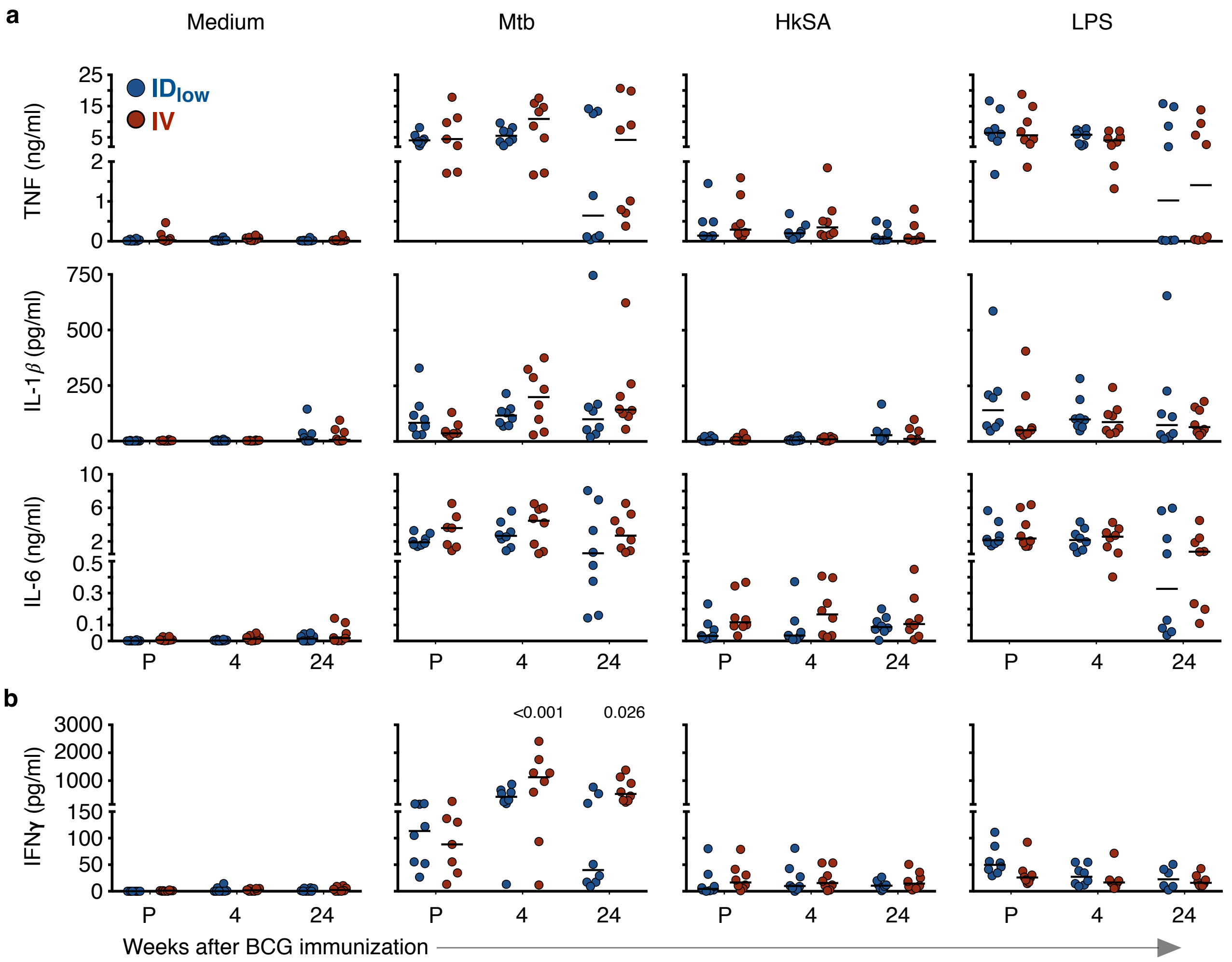

Supplementary Data 3: Innate cytokine production from PBMCs after BCG vaccination. Studies show that recent Mtb exposure or BCG immunization in humans leads to increased innate cytokine production following stimulation of PBMCs with unrelated microbial stimuli. This "trained innate immunity" has been associated with growth inhibition of BCG by PBMCs *in vitro,* and enhanced protection in mice after IV BCG vaccination. **a**, To investigate if a trained immune effect could be detected, PBMCs from NHP (n = 8 macaques) before BCG (pre-vax) and at 4 and 24 weeks after ID_{low}- or IV-BCG immunization were stimulated *in vitro* with medium alone, Mtb whole cell lysate (Mtb), heat-killed Staphylococcus aureus (HkSA), or LPS for 24 hours. TNF, IL-1β and IL-6 were measured as primary indicators of trained immunity; however, no increases in cytokines were observed for either vaccine group after vaccination. **b**, As a positive control for antigen-specific T cell stimulation, we measured IFNγ. Consistent with the flow cytometry data in **Fig. 1b, c**, PBMCs from IV BCG-immunized NHP secreted higher levels of IFNγ compared to ID_{low}-immunized NHP in response to Mtb at 4 and 24 weeks after BCG (2-way ANOVA; *P* values are Dunnett's multiple comparisons test.

Supplementary Data 4

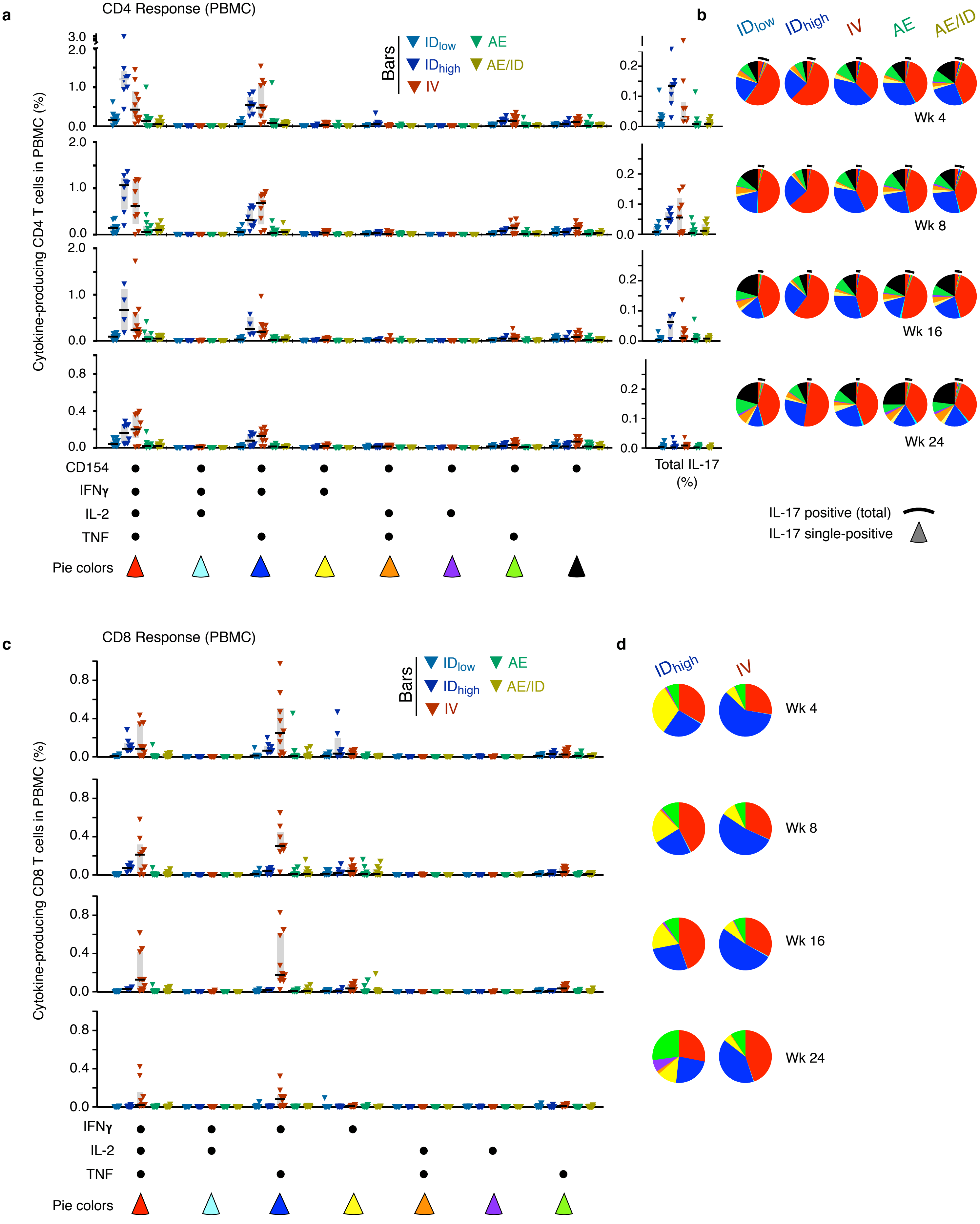

Supplementary Data 4: Quality of T cell responses in PBMCs after BCG immunization. **a**, The frequency of PPD-responsive CD4 memory T cells in PBMCs expressing CD154 with any combination of IFNγ, IL-2, or TNF, or the frequency of total IL-17 production, for macaques in each vaccine group (challenge cohorts 1–3, n = 8–11 macaques) at 4, 8, 16 and 24 weeks after BCG immunization. **b**, Pie graphs represent the proportions of total cytokine production comprising each cytokine combination as shown in (**a**), averaged for macaques in each group at each time point. Individual macaque responses are shown with interquartile range (bar) and median (horizontal line). Note that the proportion of the response producing IL-17 (with or without other cytokines) is indicated with a black arc and CD4 T cells that express CD154 (without other cytokines) are the black pie section. **c**, The frequency of PPD-responsive CD8 memory T cells in PBMCs expressing any combination of IFNγ, IL-2, or TNF in PBMCs after BCG immunization. **d**, Proportion of total cytokine response comprising each individual cytokine combination for vaccine groups that measurable CD8 T cell responses.

Supplementary Data 5

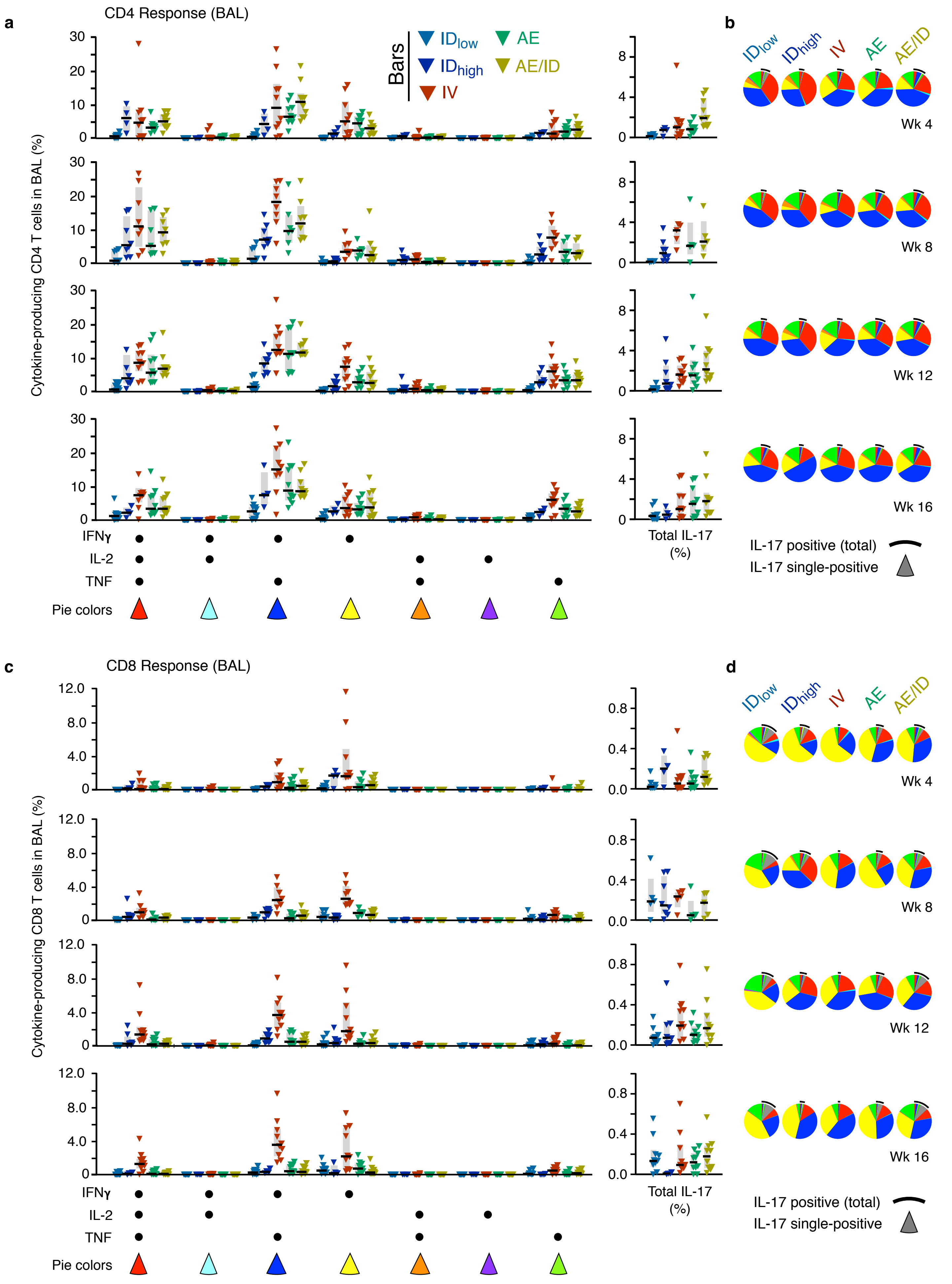

Supplementary Data 5: Quality of T cell responses in BAL after BCG immunization. **a**, The frequency of PPD-responsive CD4 memory T cells in BAL expressing any combination of IFNγ, IL-2, or TNF, or the frequency of total IL-17 production, for NHP in each vaccine group (challenge cohorts 1–3, n = 8–11 macaques) at 4, 8, 12 and 16 weeks after BCG immunization (per protocol, BAL was not sampled within 8 weeks of Mtb challenge). Individual macaque responses are shown with interquartile range (bar) and median (horizontal line). **b**, The proportion of total cytokine production comprising each cytokine combination as shown in (**a**) averaged for macaques in each group at each time point. Note that the proportion of the response producing IL-17 (with or without other cytokines) is indicated with a black arc and IL-17-single positive cells are represented by the grey pie section. CD154 expression was not measured in the BAL. **c**, **d**, The frequency and proportion CD8 T cell responses following PPD stimulation as described above.

Supplementary Data 6

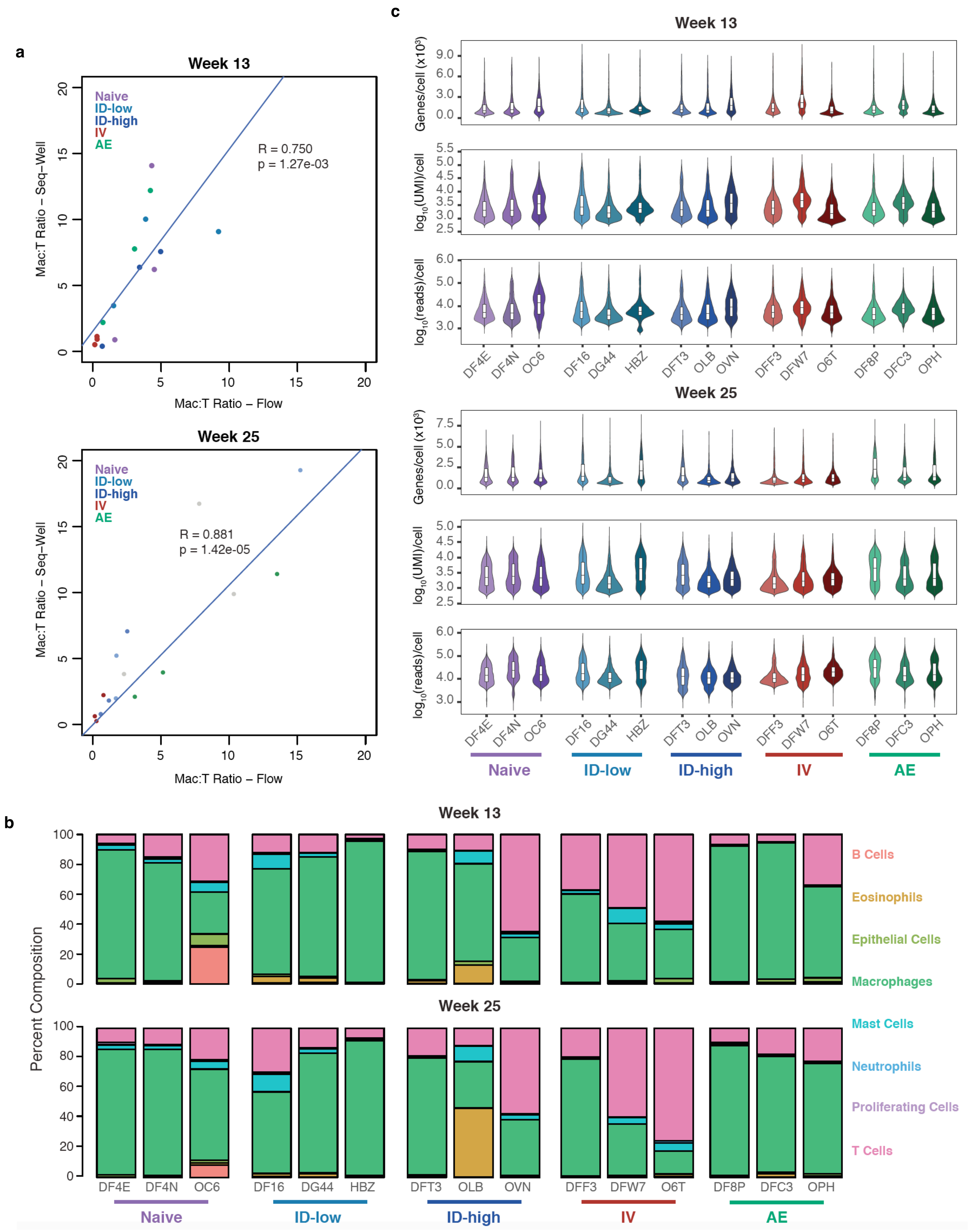

Supplementary Data 6: Data quality metrics for single-cell mRNA sequencing (Seq-Well). **a**, Scatterplots showing the relationship between the ratios of macrophages (Mac) to T cells (T) in BAL measured by flow cytometry and Seq-Well at week 13 (top) and week 25 (bottom) after BCG immunization. Data points are individual macaques (cohort 4, n = 3 macaques), color-coded by vaccine. At both time points, a significant correlation was observed between the ratio of macrophages to T cells in BAL between flow cytometric analysis (**Extended Data Fig. 3a**) and single-cell RNA-sequencing (Week 13: R = 0.75, $P = 1.3 \times 10^{-3}$; Week 24: R = 0.881, $P = 1.4 \times 10^{-5}$) **b**, Stacked bar graphs showing the cellular composition of BAL (percent of each indicated cell type in all cells combined) for each macaque in each vaccine group at weeks 13 (top) and 25 (bottom) after BCG immunization. Macrophages and T cells are depicted in green and pink, respectively. **c**, For the Seq-Well analysis, number of genes, unique molecular identifiers (UMI), and reads per cell (unstimulated and stimulated cells combined) for each macaque at weeks 13 (top) and 25 (bottom) after quality filtering (**Methods**).

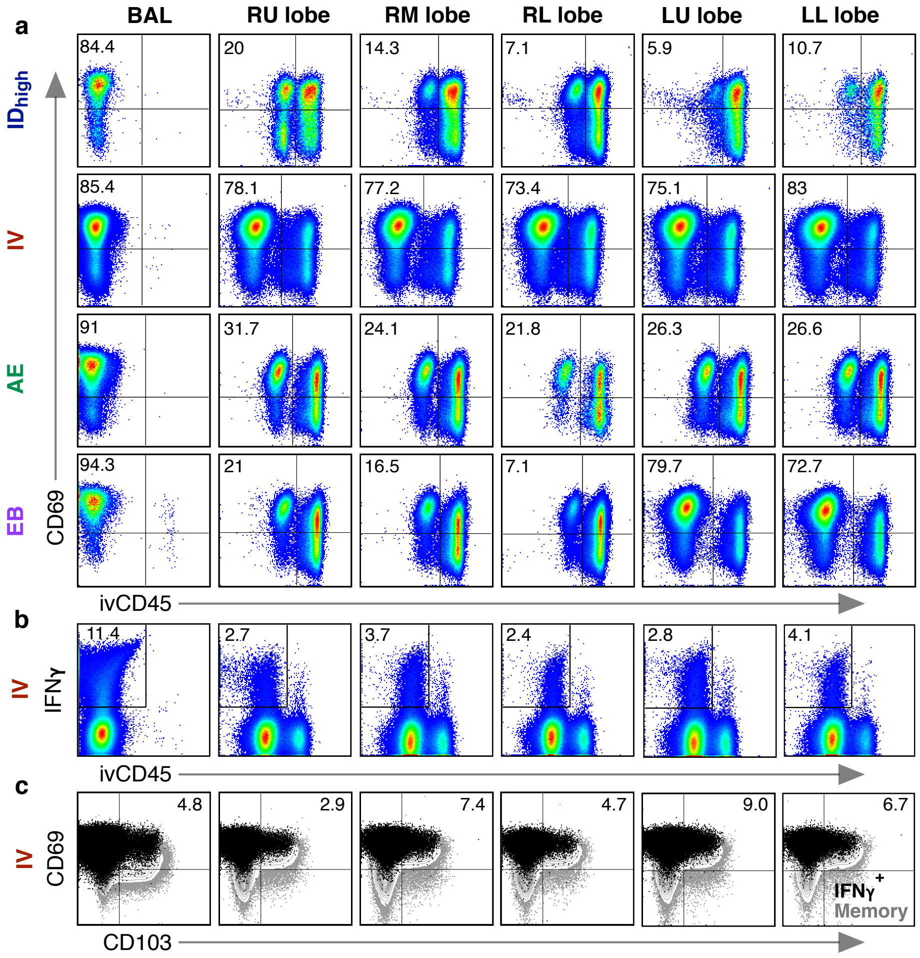

Supplementary Data 7: Detection of CD8 T cells in lung tissue following IV BCG. **a**, 1 month after BCG vaccination, tissue- versus blood-derived T cells in lung were delineated by injecting NHP IV with a fluorochrome-conjugated anti-CD45 antibody (ivCD45) prior to euthanasia, labelling leukocytes in the vasculature. Macaques (cohort 6, n = 2) received 5×10^7 CFU BCG ID, IV, AE, or endobronchially (EB) into the left lung. Cells isolated from BAL and each lung lobe were stained immediately ex vivo for surface marker expression (**a**) or stimulated with Mtb WCL and stained for intracellular cytokine expression (**b, c**), and then analyzed by flow cytometry. Shown are plots of CD8 T cells derived from the BAL and lung lobes (R, right; L, left; U, upper; M, middle; L, lower) from 1 of 2 macaques per group as shown in **Fig. 4a**. **a**, The percent of ivCD45$^-$ (unstimulated) CD8 T cells expressing the tissue-residency/activation marker CD69 for each macaque. **b**, Shown is the percent of Mtb WCL-responsive (IFNγ$^+$) CD8 T cells in the BAL and lung tissue (ivCD45$^-$) following IV BCG. **c**, IFNγ production (black) as identified in (**b**) is overlaid on total CD8 memory T cells (grey); percentages indicate the proportion of IFNγ$^+$ CD8 T cells expressing CD69 and CD103.

Supplementary Data 8

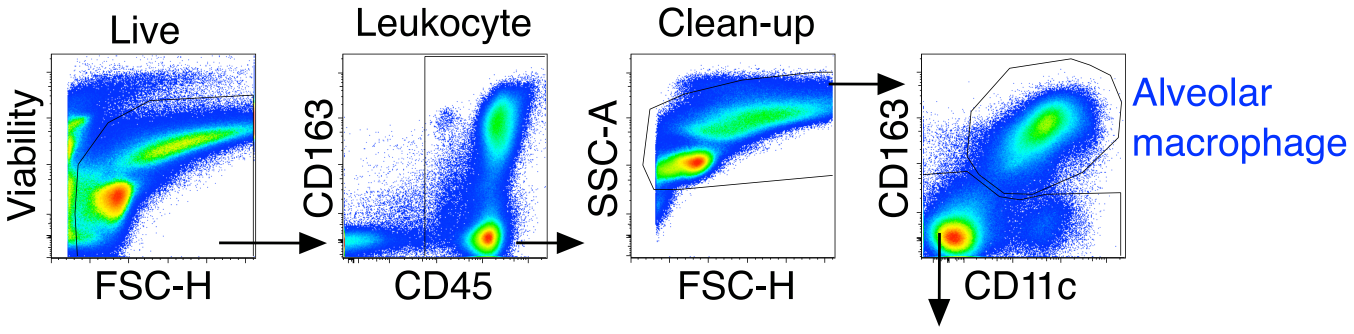

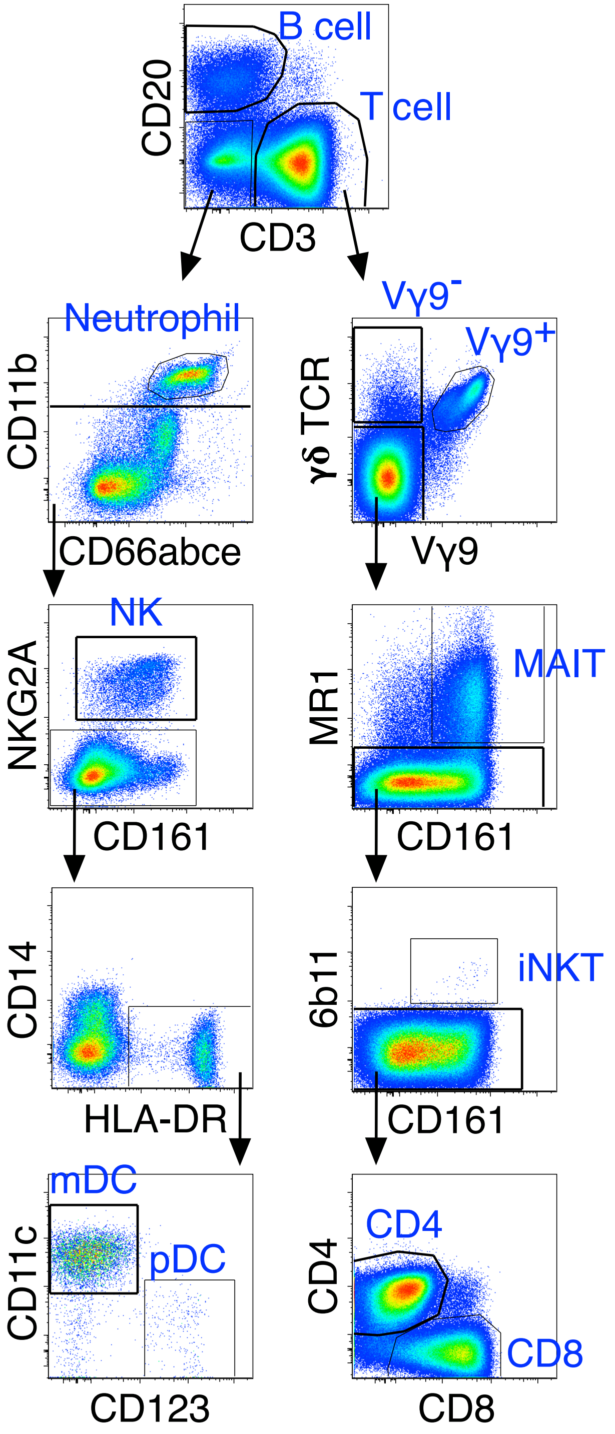

Vγ9 = Vγ9 γδ (gamma delta) T cell
NK = natural killer cell
MAIT = mucosal associated invariant T cell
mDC = myeloid dendritic cell
pDC = plasmacytoid dendritic cell
neut = neutrophil
MΦ = macrophage

Marker	Clone	Company	Fluorochrome
LIVE/DEAD	Aqua Dead Stain	ThermoFisher Scientific	
CD45	D058-1283	BD Biosciences	BV510
CD3	SP34-2	BD Biosciences	BV650
CD4	SK3	BD Biosciences	BUV737
CD8	RPA-T8	BD Biosciences	BUV395
TCR gd	5A6.E9	ThermoFisher Scientific	PE
TCR Vg9	7A5	ThermoFisher Scientific	AlexaFluor680
MR1 tetramer	Rhesus	NIH Tetramer Core	BV421
CD161	HP-3G10	BioLegend	BV605
Va24-JaQ	6b11	BD Biosciences	BV711#
CD11b	ICRF44	BioLegend	BV711#
CD69	TP1.55.3	Beckman Coulter	ECD
HLA-DR	TU36	ThermoFisher Scientific	PE-Cy5.5
CD163	GHI/61	BioLegend	APC-Cy7
CD14	M5E2	BioLegend	BV785
CD16	3G8	BD Biosciences	BUV496
NKG2A	Z199	Beckman Coulter	APC
CD20	2H7	BioLegend	BV570
CD66abce	TET2	Miltenyi Biotec	FITC
CD11c	3.9	BioLegend	PE-Cy7
CD123	6H6	BioLegend	PE-Cy5

Supplementary Data 8: Antibody panel and gating strategy used to analyze cellular composition of BAL and PBMCs after BCG vaccination. The gating strategy shown was used to determine the frequencies of leukocyte populations in the BAL and PBMCs before and after BCG vaccination, as in **Fig. 1a**, **Supplementary Data 2b**, and **Extended Data Fig. 3**. Unstimulated cells were stained for viability and then surface-stained with remaining antibodies listed. A generous "live" gate was applied to include auto-fluorescent alveolar macrophages. $CD45^+$ leukocytes were gated first, followed by $CD163^+CD11c^+$ alveolar macrophages. From non-macrophages, $CD20^+$ B cells, $CD3^+$ T cells, and non-B, non-T cells were gated. T cells were further delineated as $V\gamma9^+$ $\gamma\delta^+$ or $V\gamma9^-$ $\gamma\delta^+$ T cells, $CD161^+MR1^+$ MAIT cells, $CD161^+6b11^+$ iNKT cells (observed rarely in NHP BAL), and $CD4^+$ or $CD8^+$ T cells. Neutrophils ($CD66abce^+CD11b^+$) and NK cells ($NKG2A^+$) were gated from non-B, non-T cells. $HLA\text{-}DR^+$ cells were then gated as either $CD11c^+$ myeloid dendritic cells (mDC) or plasmacytoid dendritic cells (pDC). In some samples, CD66abce alone was used to define neutrophils and we cannot rule out eosinophil contamination. # indicates different markers conjugated to the same fluorochrome but used at different time points.

Supplementary Data 9

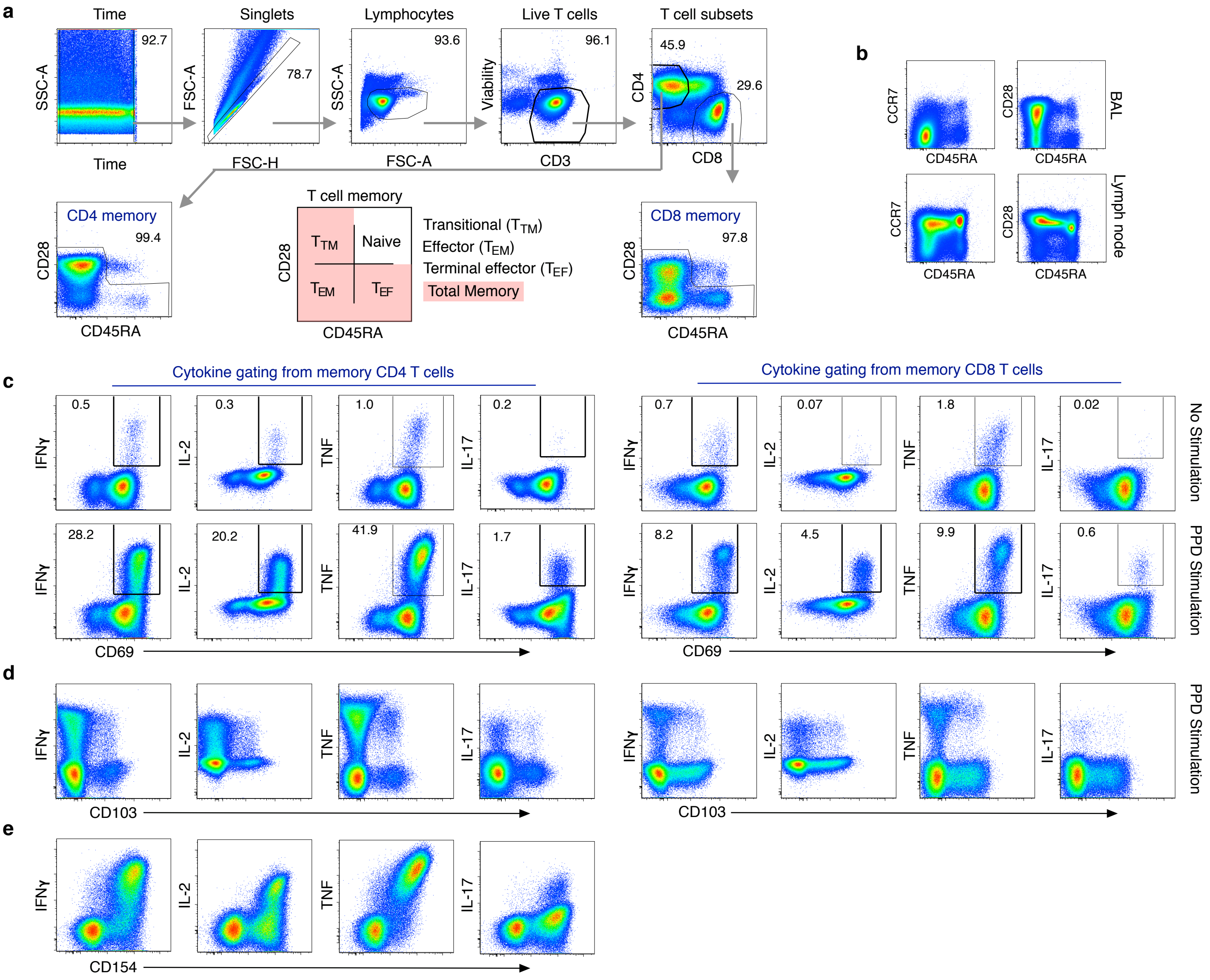

f

Marker	Clone	Company	Fluorochrome
LIVE/DEAD	Aqua Dead Cell Stain	ThermoFisher Scientific	
CD3	SP34-2	BD Biosciences	ACP-Cy7
CD4	S3.5	ThermoFisher Scientific	PE-Cy5.5
CD8	RPA-T8	BioLegend	BV570
CD28	CD28.2	BD Biosciences	PE-Cy5
CD45RA	L48	BD Biosciences	PE-Cy7
CD69	TP1.55.3	Beckman Coulter	ECD
CD103	2G5	Beckman Coulter	Alexa Fluor 680
CXCR3	1C6/CXCR3	BD Biosciences	BV711
(CXCR5)	MU5UBEE	ThermoFisher Scientific	PE
CCR6	11A9	BD Biosciences	BUV496
PD-1	EH12.2H7	BioLegend	BV785
IL-2	MQ1-17H12	BD Biosciences	BV750
IL-17A	BL168	BioLegend	BV605
TNF	Mab11	BD Biosciences	BV650
IFN-g	B27	BD Biosciences	APC
IL-21	3A3-N2.1	BD Biosciences	BV421
(CD154)	TRAP-1	BD Biosciences	BV421
(CCR7)	3D12	BD Biosciences	BB700
(IL-10)	JES3-9D7	BD Biosciences	PE
(IL-4)	MP4-25D2	BD Biosciences	PE

Supplementary Data 9: Antibody panel and gating strategy used to measure antigen-responsive CD4 and CD8 T cells in BAL after BCG immunization. The flow cytometric gating strategy shown was applied to BAL (or tissue) samples following in vitro stimulation with antigen and intracellular cytokine staining, as in (**Fig. 1d, e**, **Extended Data Fig. 4c, d**, and **Supplementary Data 5**). **a**, A narrow singlet gate excludes many alveolar macrophages; live $CD3^+$ T cells were gated using FSC-A by SSC-A, followed by exclusion of dead cells. $CD4^+$ or $CD8^+$ T cells were identified, followed by gating for memory T cells. We use CD28 and CD45RA to identify total memory in BAL although few naïve ($CD28^+CD45RA^+$) T cells are present. Memory populations in the BAL were classified as transitional memory (T_{TM}; $CD28^+CD45RA^–$), effector memory (T_{EM}; $CD28^-CD45RA^–$), or terminal effectors (T_{EF}; $CD28^–CD45RA^+$). As BAL T cells are largely negative for CCR7 staining (**b**) compared to CCR7 staining of lymph node from the same macaque), we did not apply a gate to distinguish central memory T cells (T_{CM}; $CD28^+CD45RA^–CCR7^+$) from T_{TM} ($CD28^+CD45RA^–CCR7^–$). **c**, From total CD4 or CD8 memory, IFNγ, IL-2, TNF or IL-17 production was gated against the activation marker, CD69. Shown is a representative BAL sample from an IV BCG-immunized NHP, after no stimulation or PPD stimulation; it is common to see 'background' cytokine production from unstimulated NHP BAL. **d**, Typical expression of CD103 on BAL CD4 and CD8 T cells, shown against cytokines from a PPD-stimulated sample. **e**, CD154 was included in the intracellular staining mix of some, but not all, BAL samples; CD154 was largely co-expressed with IFNγ and TNF. **f**, Antibodies used in panel; markers in parenthesis were included at some, but not all time points. In a limited number of samples, antigen-induced IL-10 or IL-4 production was tested but not detected in the BAL. For ivCD45-injected macaques, IFNγ clone B27 conjugated to BB700 (BD Bioscience) was used.

Supplementary Data 10

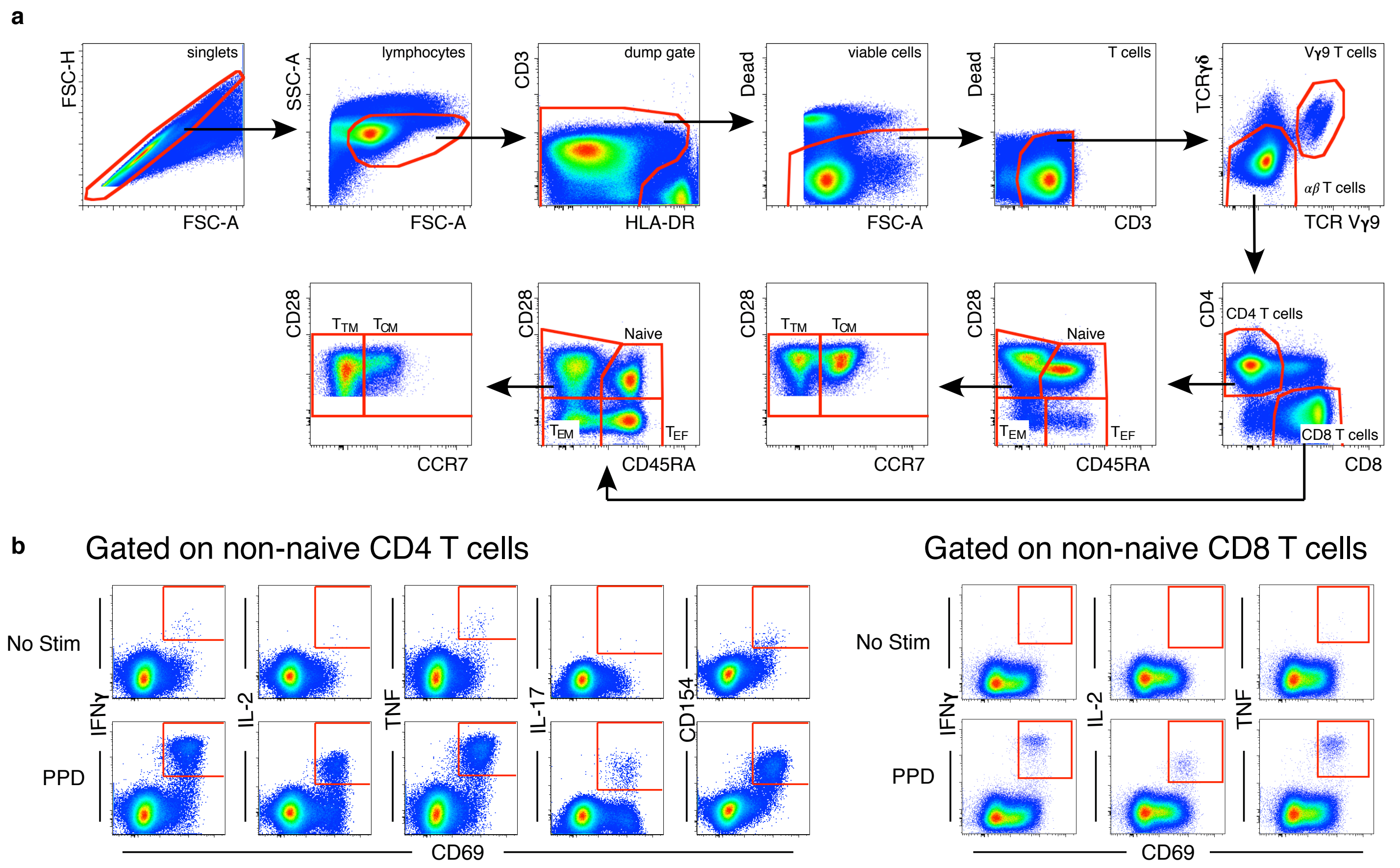

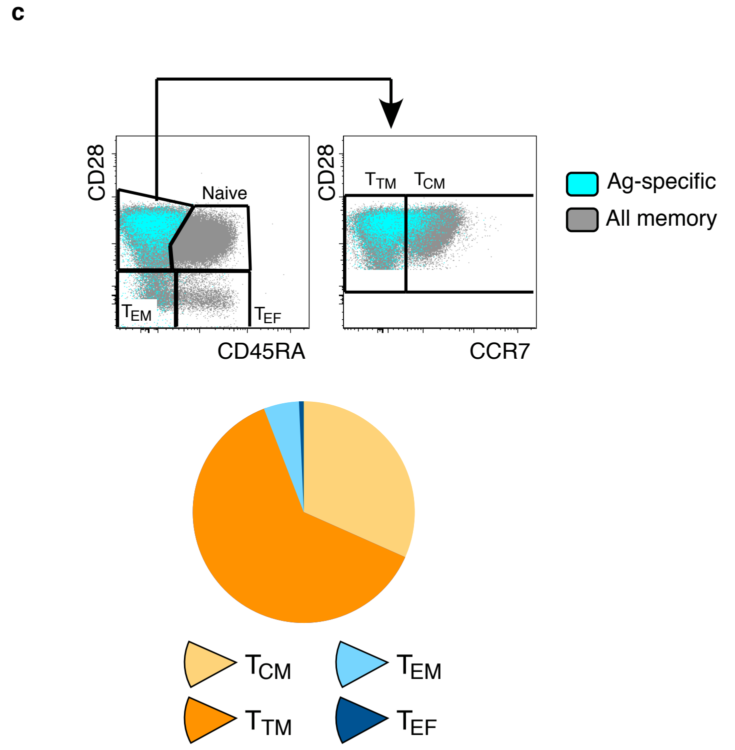

d

PBMC ICS Panel			
**Marker**	**Color**	**Clone**	**Company**
CCR4	BV510	L291H4	BioLegend
CCR6	BUV737	11A9	BD Biosciences
CCR7	AF680	150503	VRC
CD127	BV785	A019D5	BioLegend
CD137	BUV395	4B4-1	BD Biosciences
CD154	BV421	TRAP1	BD Biosciences
CD25	BUV563	2A3	BD Biosciences
CD28	PE/Cy5	CD28.2	BD Biosciences
CD3	APC/Cy7	SP34-2	BD Biosciences
CD4	BUV496	SK3	BD Biosciences
CD45RA	PE/Cy7	5H9	BD Biosciences
CD69	ECD	TP1.55.3	Beckman Coulter
CD8	BUV805	SK1	BD Biosciences
CXCR3	BV711	G025H7	BioLegend
CXCR5	SB600	MU5UBEE	Invitrogen
HLA-DR	PE/Cy5.5	Tu36	ThermoFisher
IFNg	APC	B27	BD Biosciences
IL-17A	BV570	BL168	BioLegend
IL-2	BV750	MQ1-17H12	BD Biosciences
PD-1	BB660	EH12.1	BD Biosciences
TCRγδ	PE	5A6.E9	Invitrogen
TCR Vg9	FITC	7A5	Invitrogen
TNFa	BV650	Mab11	BD Biosciences
Viability	UV-Blue	-	Invitrogen

Supplementary Data 10: Antibody panel and gating strategy used to measure antigen-responsive CD4 and CD8 T cells in PBMCs after BCG immunization. The flow cytometric gating strategy was applied to batch-analyzed PBMCs following antigen-stimulation and intracellular cytokine staining to assess the frequency and quality of cytokine producing T cells and the memory phenotype of the response, as in **Fig. 1b, c**, **Extended Data Fig. 4a, b, e, f**, and **Supplementary Data 4**. **a**, Total PBMCs were gated for singlets, followed by a lymphocyte gate (SSC-A by FSC-A), a dump gate to remove HLA-DR high cells, and a viability gate to exclude dead cells. $CD3^+$ T cells gated as $V\gamma 9^+$ $\gamma\delta^+$ T cells or $\gamma\delta^-$ $CD4^+$ and $CD8^+$ T cells. For each T cell subset, naïve ($CD28^+CD45RA^+$) cells were excluded and effector molecules were gated on total memory cells. **b**, Shown is a representative PBMCs sample from an IV BCG-immunized macaque with or without PPD stimulation. IFNγ, IL-2, TNF, IL-17 and CD154 were gated against the activation marker, CD69. **c**, The memory phenotype of the antigen-responsive T cell response was determined by gating on total CD4 or CD8 T cells and applying those gates to the Boolean-gated population of T cells producing any combination of cytokines measured. Pie charts display the proportion of the response that are central memory (T_{CM}; $CD28^+CD45RA^-CCR7^+$), transitional memory (T_{TM}; $CD28^+CD45RA^-CCR7^-$), effector memory (T_{EM}; $CD28^-CD45RA^-$), or terminal effectors (T_{EF}; $CD28^-CD45RA^+$). **d**, Antibodies used in panel.

a

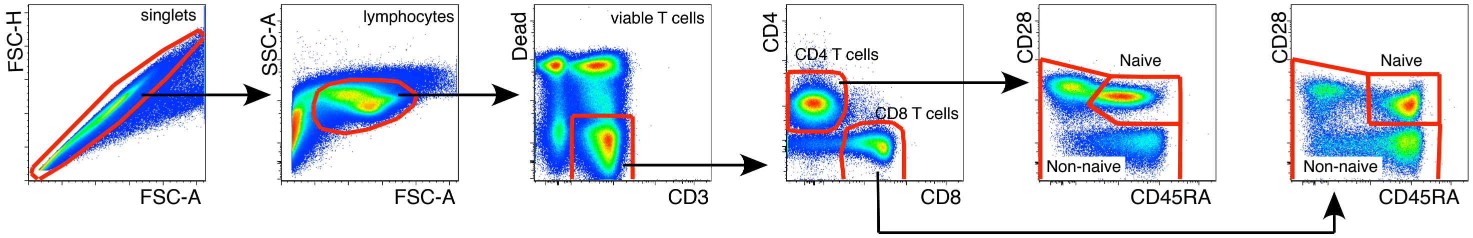

b

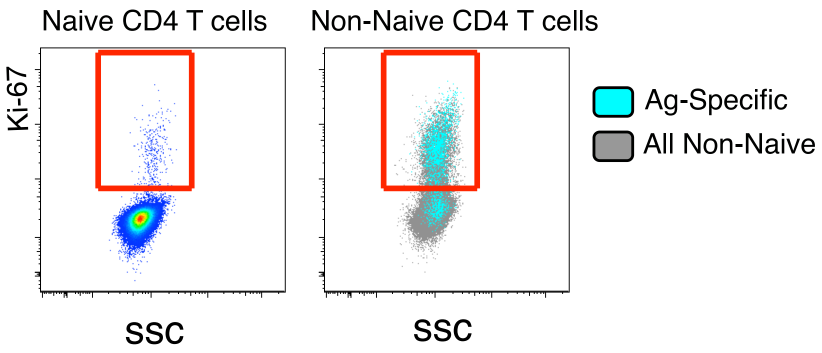

c

Ki-67 Panel			
**Marker**	**Color**	**Clone**	**Company**
CCR7	BV510	G043H7	BioLegend
CD153	PE	116614	R&D
CD154	BV421	TRAP1	BD Biosciences
CD28	PE/Cy5	CD28.2	BD Biosciences
CD3	APC/Cy7	SP34-2	BD Biosciences
CD4	BUV496	SK3	BD Biosciences
CD45RA	BV711	5H9	BD Biosciences
CD8	BUV805	RPA-T8	BD Biosciences
IFNg	APC	B27	BD Biosciences
IL-17a	BV570	BL168	BioLegend
IL-2	BV750	MQ1-17H12	BD Biosciences
Ki67	FITC	Ki67	BD Biosciences
Tbet	PE/Cy7	4B10	BioLegend
TNF	BV650	Mab11	BD Biosciences

Supplementary Data 11: Antibody panel and gating strategy to identify Ki-67-positive antigen-responsive CD4 and CD8 T cells in PBMCs after BCG immunization. The flow cytometric gating strategy was applied to PBMCs to identify the proportion of antigen-responsive CD4 and CD8 T cells that express the marker of proliferation, Ki-67, as in **Extended Data Fig. 4g–j**. As described in the methods, a multi-step staining protocol was used to optimize cytoplasmic cytokine and nuclear Ki-67 detection after in vitro antigen stimulation. Shown is a representative PBMC sample from an IV BCG-immunized NHP. **a**, Gating for cytokine-positive memory CD4 and CD8 T cells is shown in **Supplementary Data 10**. **b**, Ki-67 was gated on total CD4 or CD8 memory cells (all non-naïve) compared to naïve T cells and then the Ki-67$^+$ gate was applied to all antigen-responsive T cells. **c**, Antibodies used in panel.

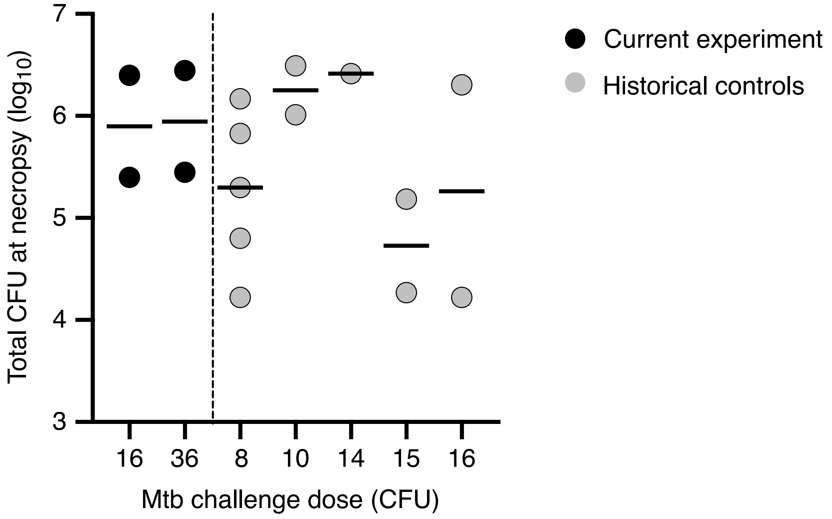

Supplementary Data 12. Disease severity in unvaccinated rhesus macaques across Mtb challenge doses. Total Mtb CFU (log_{10}) following Mtb challenge of unvaccinated rhesus macaques in this study (black dots, n = 4) is similar to that reported in our previous study (grey dots, n = 12), with infection doses ranging from 8–16 CFU.
