# Supplementary material for: Prevention of tuberculosis in macaques after intravenous BCG immunization
Source: Nature. 2020 Jan 1;577(7788):95–102. doi: 10.1038/s41586-019-1817-8 (PMC7015856; doi:10.1038/s41586-019-1817-8)
Supplement: Supplementary file 2 — Reporting Summary [file 41586_2019_1817_MOESM2_ESM.pdf]

nature research

Corresponding author(s): Robert Seder

Last updated by author(s): Oct 25, 2019

Reporting Summary

Nature Research wishes to improve the reproducibility of the work that we publish. This form provides structure for consistency and transparency in reporting. For further information on Nature Research policies, see Authors & Referees and the Editorial Policy Checklist.

Statistics

For all statistical analyses, confirm that the following items are present in the figure legend, table legend, main text, or Methods section.

n/a	Confirmed	
☐	☒	The exact sample size (*n*) for each experimental group/condition, given as a discrete number and unit of measurement
☐	☒	A statement on whether measurements were taken from distinct samples or whether the same sample was measured repeatedly
☐	☒	The statistical test(s) used AND whether they are one- or two-sided<br>*Only common tests should be described solely by name; describe more complex techniques in the Methods section.*
☐	☒	A description of all covariates tested
☐	☒	A description of any assumptions or corrections, such as tests of normality and adjustment for multiple comparisons
☐	☒	A full description of the statistical parameters including central tendency (e.g. means) or other basic estimates (e.g. regression coefficient) AND variation (e.g. standard deviation) or associated estimates of uncertainty (e.g. confidence intervals)
☐	☒	For null hypothesis testing, the test statistic (e.g. *F*, *t*, *r*) with confidence intervals, effect sizes, degrees of freedom and *P* value noted<br>*Give P values as exact values whenever suitable.*
☒	☐	For Bayesian analysis, information on the choice of priors and Markov chain Monte Carlo settings
☒	☐	For hierarchical and complex designs, identification of the appropriate level for tests and full reporting of outcomes
☐	☒	Estimates of effect sizes (e.g. Cohen's *d*, Pearson's *r*), indicating how they were calculated

Our web collection on statistics for biologists contains articles on many of the points above.

Software and code

Policy information about availability of computer code

Data collection	DiVa v7; OsiriX MD v.10.0.1
Data analysis	FlowJo v9.9.6; JMP v13; Prism v6, Excel v16.25, Pestle v1.6; SPICE v5.0; R

For manuscripts utilizing custom algorithms or software that are central to the research but not yet described in published literature, software must be made available to editors/reviewers. We strongly encourage code deposition in a community repository (e.g. GitHub). See the Nature Research guidelines for submitting code & software for further information.

Data

Policy information about availability of data

All manuscripts must include a data availability statement. This statement should provide the following information, where applicable:

- Accession codes, unique identifiers, or web links for publicly available datasets
- A list of figures that have associated raw data
- A description of any restrictions on data availability

See Mss Lines 825-835

Field-specific reporting

Please select the one below that is the best fit for your research. If you are not sure, read the appropriate sections before making your selection.

☒ Life sciences ☐ Behavioural & social sciences ☐ Ecological, evolutionary & environmental sciences

For a reference copy of the document with all sections, see nature.com/documents/nr-reporting-summary-flat.pdf

Life sciences study design

All studies must disclose on these points even when the disclosure is negative.

Sample size	See Mss Lines 869-880 (M&M, "Animals")
Data exclusions	No data excluded
Replication	See Extended Data Figure 1
Randomization	See Mss Lines 883-885 (M&M "BCG Vaccination")
Blinding	Pathologists performing the necropsies, analysts reading PET-CT scans, and technicians processing tissues were blinded from treatment group

Reporting for specific materials, systems and methods

We require information from authors about some types of materials, experimental systems and methods used in many studies. Here, indicate whether each material, system or method listed is relevant to your study. If you are not sure if a list item applies to your research, read the appropriate section before selecting a response.

Materials & experimental systems

n/a	Involved in the study	
☐	☒	Antibodies
☒	☐	Eukaryotic cell lines
☒	☐	Palaeontology
☐	☒	Animals and other organisms
☒	☐	Human research participants
☒	☐	Clinical data

Methods

n/a	Involved in the study	
☒	☐	ChIP-seq
☐	☒	Flow cytometry
☒	☐	MRI-based neuroimaging

Antibodies

Antibodies used	See Suppl Data 8-11
Validation	All monoclonal antibodies used for flow cytometry were titrated to obtain optimal signal to noise, comparing the specific staining to a gold standard or previously published monoclonal antibody to ensure the staining is biologically appropriate.

Animals and other organisms

Policy information about studies involving animals; ARRIVE guidelines recommended for reporting animal research

Laboratory animals	See Mss Lines 857-867 (M&M, "Animals")
Wild animals	N/A
Field-collected samples	N/A
Ethics oversight	See Mss Lines 857-867 (M&M, "Animals")

Note that full information on the approval of the study protocol must also be provided in the manuscript.

Flow Cytometry

Plots

Confirm that:

☐ The axis labels state the marker and fluorochrome used (e.g. CD4-FITC).

☒ The axis scales are clearly visible. Include numbers along axes only for bottom left plot of group (a 'group' is an analysis of identical markers).

☒ All plots are contour plots with outliers or pseudocolor plots.

☒ A numerical value for number of cells or percentage (with statistics) is provided.

Methodology

Sample preparation	See mss Lines 972-1002 (M&M Multiparameter Flow Cytometry)
Instrument	BD LSR II and BD Symphony A5
Software	Diva v7 and FlowJo v9.9.6
Cell population abundance	N/A
Gating strategy	See Supplemental data 8-11

☒ Tick this box to confirm that a figure exemplifying the gating strategy is provided in the Supplementary Information.
